# Off-target autophagy disruption associated with a novel liver toxicity in dogs for a highly basic heterobifunctional protein degrader

**DOI:** 10.3389/fphar.2025.1664889

**Published:** 2025-12-10

**Authors:** James E. Kath, Rebecca Kohnken, Timothy Brayman, Junhai Yang, Katharina Haag, Jingyi Gong, Christopher Untucht, Christiane Imhof, Kenneth P. Robinson, Ryan A. McClure, James W. Sawicki, Alexander Buzenski, Rita Ciurlionis, Asra Habibullah, Bo Liu, Christian Pohl, Zhaozhong Jia, Spencer O. Scholz, Lise I. Loberg, Alexey Rivkin

**Affiliations:** 1 Development Biological Sciences, AbbVie Inc., North Chicago, IL, United States; 2 Neuroscience Discovery, AbbVie Deutschland GmbH & Co KG, Ludwigshafen, Germany; 3 Small Molecule Therapeutics and Platform Technologies, AbbVie Inc., North Chicago, IL, United States; 4 Global Medicinal Chemistry, AbbVie Inc., South San Francisco, CA, United States

**Keywords:** degrader, bile duct, cholangiocyte, pKa, phospholipidosis

## Abstract

**Introduction:**

The observation of hepatobiliary toxicity in a repeat-dose Good Laboratory Practice-compliant dog toxicology study was a primary driver for the deprioritization of a preclinical heterobifunctional protein degrader candidate, Compound X. The pathology of large bile duct epithelial hyperplasia was novel and its pathogenesis unknown.

**Methods:**

In this study, a thorough characterization and mechanistic investigation are presented with both short-term exploratory animal studies and *in vitro* recapitulation. Cholangiocytes, epithelial cells lining bile ducts, were the toxicity target, with an accumulation of Compound X in both bile and the affected cells.

**Results:**

Proteome profiling and high-content imaging highlighted a significant disruption to autophagy, with a dramatic increase in autophagosomes. A whole genome CRISPR-Cas9 screen identified the lysosomal V-ATPase as a key mediator of cell sensitivity to Compound X. This was further demonstrated by a rescue of toxicity *in vitro* by the V-ATPase inhibitor, bafilomycin A1, directly linking the pathology to disruption of the autophagy-lysosome system. Importantly, neither the degradation target of Compound X nor the E3 ligase it recruits, CRBN, were similarly implicated. An analog degrader with differentiated physicochemical properties, most notably a reduced pKa, was identified with significantly reduced hepatobiliary toxicity despite similar bile concentration. Together, these data indicate that uptake of the large, basic, and lipophilic Compound X into cholangiocyte lysosomes drives a unique bile duct pathology.

**Discussion:**

This mechanism is a further demonstration of how the physicochemical properties of bifunctional degraders may challenge preclinical development, and its elucidation provides a path forward for development of degrader compounds with improved toxicity profiles.

## Introduction

Heterobifunctional protein degraders belong to a new and growing class of small molecule therapeutics that direct the destruction of target molecules rather than inhibiting or otherwise modulating an enzymatic function. Degraders contain two pharmacophores that respectively bind to the therapeutic target (target-binding ligand), and an E3 ubiquitin protease ligase, such as cereblon (CRBN) or the Von Hippel-Lindau tumor suppressor (VHL) (ligase-binding ligand). An optimized linker connecting the two chemical groups orients the target for ubiquitination and trafficking to the ubiquitin-proteasome system. Potential advantages of targeted protein degradation include the conversion of “functionally neutral” target binders into degraders with potential therapeutic benefit, the targeting of “undruggable” targets, eliminating scaffolding functions of proteins leading to differentiated efficacy, a potential for catalytic activity leading to sub-stoichiometric potency, and increased selectivity between close homologs (Békés et al., 2022; Bondeson et al., 2015; Lynch et al., 2024; Cantley et al., 2022).

Since the first clinical stage degraders were disclosed in 2019, clinical trials have been announced for more than 20 unique degrader assets, reflecting the therapeutic promise of this modality. This promise has been balanced by several challenges in preclinical development. Industry surveys conducted by the International Consortium for Innovation and Quality in Pharmaceutical Development (IQ Consortium) highlighted safety considerations including delayed toxicity due to the kinetics of degradation and target resynthesis, undesired degradation of off-targets of the target-binding pharmacophore, and for CRBN-recruiting degraders, degradation of neosubstrates such as SALL4, which is linked to the teratogenicity of imides, most notably thalidomide (Hemkens et al., 2023).

The physicochemical properties of degraders are also considered a liability for preclinical development. As a class, bifunctional protein degraders are larger (molecular weight significantly greater than 500 Da) and more lipophilic (calculated octanol-water partition coefficient, or ClogP >5) than most approved orally administered drugs, constituting a “beyond rule of five” chemical space (Poongavanam and Kihlberg, 2021; Doak et al., 2014). High molecular weight and lipophilic small molecules are generally (but not universally) less soluble in aqueous solution, less permeable across cell membranes, and poorly absorbed into systemic circulation after oral administration (Volak et al., 2023). Physicochemical properties can also be associated with phospholipidosis (PLD), a toxicologic finding commonly observed for cationic amphiphilic drugs–those with both a high (basic) acid dissociation constant (pKa) and high ClogP (Ploemen et al., 2004), and this has been reported for degraders (Hemkens et al., 2023). Although several orally bioavailable bifunctional protein degraders have been reported (He et al., 2024; Rej et al., 2024; Kofink et al., 2022; Zeng et al., 2023; Apprato et al., 2024), the physicochemical properties of this chemical space presents a major development challenge. There are, however, limited descriptions of degrader-specific preclinical toxicity in the literature to-date, and potential toxicities relating to the unique physicochemical properties of degraders beyond PLD remain poorly understood.

Compound X, a late preclinical-stage degrader developed to direct the ubiquitination and proteosome degradation of an undisclosed kinase target, resulted in adverse, non-monitorable, and irreversible large bile duct toxicity when administered to dogs for 4 weeks. The finding was novel, and the mechanism was unknown, particularly given the lack of target expression in the affected tissue. We hypothesized that Compound X was intrinsically cytotoxic to the bile duct and set out to further investigate and characterize this toxicity.

Here we report a novel hepatobiliary toxicity for an preclinical degrader candidate that is mechanistically related to its physicochemical properties, cellular accumulation, and disruptions to the autophagy-lysosome pathway, rather than its target, off-targets, or CRBN recruitment. While this study further highlights the potential challenges of the physicochemical space occupied by protein degraders, it also provides an example of how diverse scientific approaches can be used in concert to provide mechanistic detail and correlates of this toxicity, which can then be used to effectively screen and identify related compounds with significantly reduced toxicity.

## Materials and methods

### Compounds

Heterobifunctional bifunctional protein degraders (Compound X, Y, Z, and non-degrading control Compound A) were designed and synthesized at AbbVie, Inc. Identity and purity (>95% for all) was confirmed by liquid-chromatography mass spectrometry (LC-MS). Bafilomycin A1 was purchased from Sigma as a ready-made dimethyl sulfoxide (DMSO)-formulated stock solution (part number [P/N] SML1661, 0.1 mM); chloroquine diphosphate was purchased from abcam (P/N ab142116) and solubilized in sterile water for a 100 mM stock solution.

### Animal use

All protocols were approved by the AbbVie Institutional Animal Care and Use Committee (IACUC) and performed in accordance with the National Institutes of Health Guide for the Care and Use of Laboratory Animals. All animals were housed under standard laboratory conditions with a 12-h light/dark cycle, in a temperature and humidity-controlled room with free access to standard beagle diet and water.

### Repeat-dose toxicology studies

In the Good Laboratory Practices (GLP)-compliant toxicology study, Compound X was administered daily by oral capsule to purpose-bred beagle dogs (n = 3 per sex per group) for at least 28 consecutive days, with a subset of animals (n = 2 per sex per group) in the control and high dose groups continuing for a 28-day recovery period. Dosages administered were 0 (10/30/60 EtOH/PEG400/Phosal 53 MCT), 3, 10, and 30 mg/kg/day at a dose volume of 1 mL/kg. A 14-day non-GLP study was conducted in a similar manner with n = 1 per sex per group at the same doses.

In the exploratory toxicology study, Compound X was administered daily by oral capsule to beagle dogs (n = 2 males per group) for 5 consecutive days at 0 (vehicle as above) and 30 mg/kg/day. Analog degraders with differentiated physicochemical properties but similar pharmacological properties were also evaluated for biliary toxicity in dogs. Compound Y, formulated in 10/30/10/50 EtOH/PEG400/Kolliphor RH40/Phosal 50 PG, was administered by oral gavage to beagle dogs (n = 1 male and 1 female) at 50 mg/kg/day for 5 consecutive days. Compound Z was administered in the same vehicle by oral gavage to n = 2 female beagle dogs at 50 mg/kg/day for 5 consecutive days. All formulations were administered at a dose volume of 1 mL/kg.

Clinical pathology (hematology, clinical chemistry, coagulation) was collected at the end of each toxicology study in addition to whole blood for plasma toxicokinetic analysis. Hematology and clinical chemistry samples were analyzed using the ADVIA® 2120 automated hematology analyzer and the Abbott Architect c16000, respectively.

At the end of each toxicology study, dogs were humanely euthanized by exsanguination preceded by induction of a deep plane of anesthesia by barbiturate injection. For the 5-day exploratory study, in addition to standard protocol-driven collection of major organs, bile was collected, the volume measured, and an aliquot frozen for toxicokinetic analysis. Sections of hilar liver lobes were collected and frozen for mass spectrometry imaging and toxicokinetic analysis. Sections of liver and common bile duct were collected and immersed in 10% neutral buffered formalin and trimmed, embedded in paraffin, and processed to glass slides in the standard manner and stained with hematoxylin and eosin for microscopic evaluation by an American College of Veterinary Pathologists board-certified pathologist.

#### Immunohistochemistry, special stains, and electron microscopy

Immunohistochemical (IHC) staining was performed on 5 μm-thick formalin-fixed paraffin-embedded histologic sections. Primary antibodies for Ki-67 were obtained from Invitrogen (Cat #PA5-19462, Carlsbad CA United States), and for LC3B from Novus Biologicals (Cat #NB100-2220, Centennial CO United States). Chromagen-based IHC stains were performed using the BOND Rx Automated Research Stainer (Leica Biosystems, Buffalo Grove IL United States). All IHC slides were counterstained with hematoxylin, dehydrated in graded alcohols, cleared with xylene, and coverslipped.

Masson’s Trichrome staining was performed on 5 μm-thick formalin-fixed paraffin-embedded histologic sections. Masson’s was performed manually using Newcomer Supply Kit part #9179B (Middelton WI United States).

For ultrastructural imaging, tissues were removed from initial fixative and trimmed into 1 × 1 × 1 mm cubes and stored in Karnovsky’s fixative for 24 h. Tissues were then serially processed in 0.1 M Sorenson’s phosphate buffer, 1% osmium tetroxide, and deionized water. Further processing in graded ethanol to dehydrate the tissues was performed. Tissues were then placed in embedding mold, topped with resin, and allowed to polymerize for 3 days at 60 °C. Sections were cut using a Leica EM ultramicrotome (Wetzlar Germany) to 1 μm and stained with Toluidine blue. Grids were prepared for each block by sectioning to 85 nm and stained in droplets of 4% aqueous uranyl acetate, Reynold’s lead citrate, and water. Grids were imaged on a JEM-1400 scope (JEOL, Peabody MA United States) and captured using an AMT NanoSprint 12 camera (AMT Imaging, Woburn MA United States).

#### Toxicokinetic measurement

Plasma and tissue drug concentrations were determined using protein precipitation extraction followed by liquid chromatography tandem mass spectrometry (LC-MS/MS) detection. The lower limit of quantification (LLOQ) was set at 1 ng/mL for plasma and 1,250 ng/g for liver.

#### Mass spectrometry imaging

A thorough description is provided in the Supplementary Experimental Procedures. Briefly, liver sections were coated with matrix DHA or CHCA solutions using a HTX TM sprayer M3+ (HTX Technologies, Chapel Hill NC United States). Imaging was carried out on a Bruker timsTOF flex mass spectrometer (Bruker, Billerica MA United States) with the laser set for 5–100 shots/pixel using positive ion mode.

#### Cholangiocyte cell culture and viability testing

Human primary cholangiocytes (intrahepatic biliary epithelial cells) were purchased from: ScienCell Research Laboratories (#5101, Carlsbad CA United States) along with epithelial cell medium (#4101); Applied Biological Materials Inc (#T5555, Richmond BC Canada) along with PriGrow X series medium (ABM, #TM5555); and Creative Bioarray (#CSC-C9352W, Shirley NY United States) along with human epithelial cell medium (#CM-1098X, Creative Bioarray). Cells were grown in an incubator (37 °C, 5% CO_2_) and maintained according to vendor specifications, up to 5 passages. Unless otherwise specified, data represents ScienCell primary human cholangiocytes and media.

For the cholangiocyte viability assessment, cells were seeded at 10,000/well on collagen coated white clear bottom 96 W plates (Corning #256701, Glendale AZ United States) and allowed to attach for ∼4 h. Compounds were dispersed in triplicate at half-log concentrations (0.1–100 µM) using the TECAN compound dispenser, normalizing dimethyl sulfoxide (DMSO) vehicle content to 0.1% for all wells. Plates were incubated for 48 h and then analyzed visually by microscopy for morphology effects and/or compound precipitation. After determining lowest compound concentration showing precipitation, media was aspirated and CellTiter Glo 2.0 (Promega, #G9243, Madison WI United States) was added to determine cytotoxicity. Plates were shaken for ∼2 min and allowed to sit for a minimum of 10 min for signal stabilization. Luminescence was determined using the Clariostar (BMG Labtech, Ortenberg Germany). Data were fit using GraphPad Prism, utilizing the nonlinear regression model log(inhibitor) vs. response–variable slope (four parameters), with the bottom parameter constrained to zero.

For compound uptake LC-MS, protein immunoblotting, and proteomics experiments, primary cholangiocytes (ScienCell #5101, unless specified) were seeded at 200,000/well on collagen coated white clear bottom 6-well plates (Corning #356400) and allowed to attach overnight. Cells were treated with compounds at concentrations indicated for 3 h (immunoblotting), 18 h (proteomics), or 48 h (compound uptake) using DMSO vehicle content normalized across wells, up to 0.2%. 48 h was selected for compound uptake experiments to match the cell viability assay conditions; 18 h was selected for the initial assessment of proteome-wide changes leading to and accompanying cytotoxicity; 3 h to observe pathway changes with minimal confounding effects of cytotoxicity. Immunoblotting was performed in biological duplicates, except for the single confirmatory immunoblot in Supplementary Figure S7D; proteomic samples were prepared with cells from three different vendors, each treated in technical duplicate or triplicate; for compound uptake LC-MS studies, samples were prepared in technical triplicate and repeated in biological duplicate. Following compound incubation, cells were washed twice with phosphate buffer saline (PBS) and, except for compound uptake studies, lifted off plates with Accumax (Sigma-Aldrich A7089). Cells were pelleted at 700 × g for 5 min, washed with PBS, and pellets were stored at −80 °C for later sample preparation.

Methods for the C-DILI cholestatic hepatotoxicity assay and CRBN knockdown in cholangiocytes are described in Supplementary Experimental Procedures.

#### Quantification of compound uptake into cholangiocytes by LC-MS

For quantification of compound uptake into cholangiocytes, cells were prepared and incubated with compound as described above. The following fractions for each sample were prepared for LC-MS compound quantification: (1) Input media: equivalently prepared media with compound, but not added to cells; (2) Spent media: media removed from cells after treatment (0 or 48 h); (3) Washes: the combined PBS washes of cells upon harvest. (4) Cells: remaining in each plate well after the final PBS wash was removed and saved. As the solubility of Compound X in pH 7.4 buffer is low and precipitation was visible at higher concentrations, “soluble” and “insoluble” samples were obtained for aqueous fractions #1-3 by clarifying samples at 2,800 *g* for 10 min and transferring the supernatant to a separate Eppendorf tube. Fractions from each sample were then prepared and the compound abundance per sample volume of Compounds X, Y, or Z was quantified by a Shimadzu Prominence UPLC (Shimadzu Corporation, Kyoto Japan) and AB Sciex 6500 QTrap Triple Quadrupole mass spectrometer system (Sciex, Framingham MA United States), as described in the Supplementary Experimental Procedures.

#### Immunoblotting

Cell pellets were lysed on ice for 30 min with RIPA buffer (EMD Millipore #20-188, diluted with water to 1X) supplemented with cOmplete Mini EDTA-free protease inhibitor cocktail (1X final, Roche #11836170001, Sigma Aldrich, St Louis MO United States) and benzonase (25 U/mL final). Lysate protein content was determined with the Pierce 660 nm Protein Assay Kit (Cat #22662, Thermo Fisher Scientific, Waltham MA United States). Lysate corresponding to 20 µg protein was combined with LDS Buffer (1X final, Thermo #NP0007) and 1 M dithiothreitol stock (100 mM final), heat denatured at 95 °C for 10 min, and loaded into the wells of a NuPage Bis-Tris Mini Protein Gel (Thermo #NP0321). Proteins were separated at 200 V for 30–40 min using a XCell SureLock Mini-Cell (Thermo #EI0001) and MES SDS Running Buffer (Thermo #NP0002).

Proteins were next transferred to a PVDF membrane (Thermo #IB24002) with an iBlot 2 Gel Transfer Device (Invitrogen #IB21001) using the standard 7 min method. The membrane with rinsed with PBS, blocked for 30–60 min with a 1:1 mixture of Intercept PBS Blocking Buffer (LiCor 927-70001, LiCor Biosciences, Lincoln NE United States) before probing overnight with primary antibodies (10 mL, in a 1:1 mixture of PBS with 0.1% Tween 20 [PBST] and Intercept PBS Blocking Buffer). The next day, the membrane was washed with PBST (5 min, 5 times), then probed with secondary antibodies (10 mL, in a 1:1 mixture of PBST and Intercept PBS Blocking Buffer). The membrane was finally washed five times with PBST and then imaged on a LiCor Odyssey CLx Imager.

#### LC-MS/MS analysis of cholangiocyte proteomes

Sample proteomes from Compound X or vehicle-treated cholangiocyte pellets from ScienCell (0, 0.3, 1, and 3 µM Compound X, in triplicate) were reduced, alkylated, digested to tryptic peptides, and isolated with solid phase extraction (SPE). These peptides, for individual samples, were analyzed with a timsTOF Pro 2 mass spectrometer (Bruker Daltonics) equipped with an EvoSep One liquid chromatography system using data-independent acquisition (DIA) mass spectrometry in the parallel accumulation-serial fragmentation (PASEF) mode. Sample proteomes from Compound X or vehicle-treated cholangiocyte pellets from ABM and Creative Bioarray (0, 1, and 10 μM, in duplicate for each vendor) were prepared similarly, but labeled with the tandem mass tag (TMT) isobaric labeling reagent after digestion and the 12 samples were pooled into a single sample (containing both ABM and Creative Bioarray samples). The pooled sample was then fractionated into 12 fractions and analyzed on an Orbitrap Fusion Lumos Tribrid mass spectrometer equipped with a EASYnLC 1,200 liquid chromatography system (Thermo Scientific). Additional information of sample prep and LC-MS/MS analysis are available in the Supplementary Experimental Procedures.

#### LC-MS/MS data analysis

Protein-level quantification data across samples, as outputs from either MaxQuant (v1.6.15, for TMT-labeled sample MS files) or Spectronaut (v18.6, for DIA-MS files), were analyzed with a custom R program to statistically test differences between conditions. First, protein entries corresponding to common contaminants and reverse database hits were removed, as well as those with >50% missing values across the sample set (first set: DIA data from ScienCell cholangiocyte samples, 8,120 protein groups remaining; second set: TMT data-dependent acquisition (DDA) data from ABM and Creative Bioarray cholangiocyte samples, 6,264 protein groups). Missing protein quantification values were then imputed using a normal distribution, and the protein quantification distributions were quantile-normalized. Finally, proteins were evaluated for differential abundance changes across replicates for each Compound X concentration for a given cell line against the vehicle replicate set from the same cell line. This was accomplished via linear modeling and an empirical Bayes method (Smyth, 2004) as implemented by the R package *limma* (Ritchie et al., 2015) functions *lmFit* and *eBayes*. Proteins with significant abundance changes vs. vehicle treatment (“hits”) were defined as those having: (1) at least two unique peptides identified, (2) a log2 fold-change (positive or negative) of magnitude at least 3.5-fold times the standard deviation for that condition, and (3) an adjusted *p* value <0.05). Hits identified with the highest Compound X concentration in cholangiocytes from at least two different vendors were selected for pathway enrichment analysis.

#### Autophagosome-lysosome high content imaging assay

U-2 OS cells stable expressing a N-terminal fusion of LC3B to GFP (U-2 OS GFP-LC3B) was used to quantify lipidated LC3B (LC3B-II) and autophagosomes via microscopy; LysoTracker Red DND-99 (#L7528, Thermo) was additionally used to quantify the number of lysosomes. 6,000 cells/well were seeded in a 96-well assay plate with 100 mL media. 24 h later, media with double-concentrated vehicle (0.35% final) or compound (0.01–31.6 µM final, at eight half-logarithm concentration steps) was added, with two technical replicate wells per condition. A reference compound, AZD8055 (an mTOR inhibitor that induces autophagy), was also included on the same plate, as well as 3 µM Torin-1 as a positive control for autophagy induction. Cells were incubated (37 °C, 5% CO_2_) for 3 h before fixing, staining nuclei with Hoechst 33342 dye (#H3570, Thermo), and imaging. Additional information on fixation and image acquisition are available in the Supplementary Experimental Procedures.

Mean autophagosome (GFP-LC3B) and lysosome (LysoTracker) spot numbers were normalized using high (Torin-1) and low (vehicle) conditions. Dose response curves were generated with a 4-parameter logistic model (Hill equation) and the Levenberg-Marquardt algorithm with a maximum of 50 iterations for fitting. The resulting curve was used to extract half-maximal effective concentration (EC50) endpoint. In addition, the maximum observed mean effect at a given concentration (Ymax observed shown as Max [%]) is reported. For inactive compounds with Ymax observed lower than 50% or negative curve slope, EC50 was reported as >X Max (>highest concentration measured and not excluded). For all other compound, EC50 calculated from the curve was reported. The Z′ prime calculation for each plate was additionally used for assay performance. The long-term assay stability was tracked by the moving minimum significant ratio (moving MSR) of the AZD8055 EC50.

#### Genome-wide CRISPR knockout and CRISPRa screening

A whole-genome CRISPR screen for genes that convey sensitivity or resistance to Compound X upon knockout (CRISPR KO) was performed using a stable U-2 OS Cas9 cell line and the human Brunello knockout lentiviral library (Doench et al., 2016). In parallel a whole genome CRISPR screen for genes that convey sensitivity or resistance to Compound X upon activation (CRISPR-a) was performed using a stable U-2 OS dCas9 cell line and the human Calabrese Set A activation lentiviral library (Sanson et al., 2018). Details on stable cell line generation, library titration and transduction, selection, and next-generation sequencing (NGS) library preparation and sequencing are provided in the Supplementary Experimental Procedures.

## Results

### Repeated administration of Compound X in dogs results in bile duct toxicity

**FIGURE 1:**
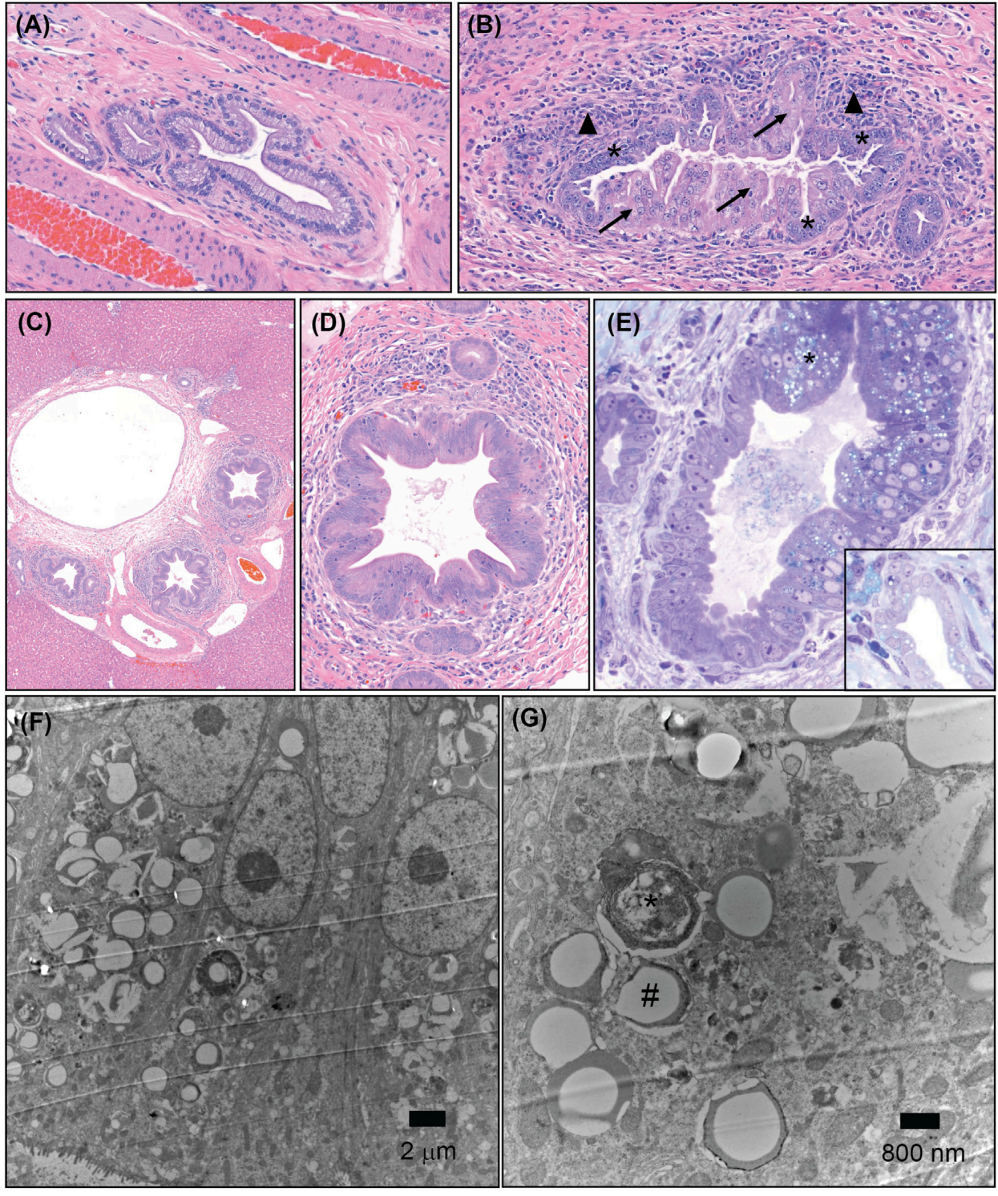
Bile duct pathology resulting from administration of Compound X in dogs. **(A)** Representative light microscopy image of a large bile duct from a vehicle control dog, ×20 magnification, Hematoxylin and eosin (HE) staining. **(B)** Degeneration (arrows), necrosis, and regeneration (asterisks) of cholangiocytes from a large bile duct associated with mixed inflammatory infiltrate (arrowheads) from a dog administered Compound X for 5 days, 20×, HE. **(C,D)** Biliary hyperplasia of a large bile duct from a dog administered Compound X for 28 days, **(C)** 5×, **(D)** 20×, HE. **(E)** Hyperplastic cholangiocytes characterized by vacuolation and accumulation of basophilic stippled material (asterisk) from a dog administered Compound X for 5 days, 20×, Toluidine blue staining. Inset: Normal bile duct from vehicle dog. **(F,G)** Ultrastructural images from a dog administered Compound X for 5 days, **(F)** 5,000×, **(G)** 20,000×. Note accumulation of autophagosomes (asterisk) and lysosomes (hashtag). Basilar aspect upper right and luminal aspect lower left in each image.

In an exploratory toxicology study in dogs with Compound X administered orally at 30 mg/kg/day for 5 days, histopathology of the liver revealed moderate to marked degeneration and necrosis limited to the epithelium (cholangiocytes) of large (intrahepatic) and extrahepatic bile ducts (see Supplementary Figure S1 for an anatomical diagram). This highly unusual finding was characterized by a spectrum of cellular swelling, hypereosinophilia, nuclear fragmentation (consistent with degeneration and necrosis), and cellular basophilia (consistent with regeneration). The luminal profile of the large ducts was tortuous with the epithelium thrown into folds, consistent with hyperplasia (confirmed by Ki67 immunohistochemistry, Supplementary Figure S2A). Surrounding affected ducts was a mixed inflammatory infiltrate composed of lymphocytes, macrophages, and neutrophils (Figures 1A,B). The hepatocellular parenchyma (including hepatocytes, small bile ductules, and vasculature) was unremarkable. Thin sections stained with Toluidine blue highlight vacuolization of cholangiocytes in affected bile ducts, as well as accumulation of basophilic stippled material (Figure 1E). Ultrastructural evaluation by electron microscopy (EM) of bile ducts from the 5-day study revealed cytosolic autophagosomes and lysosomes within injured cholangiocytes (Figures 1F,G) which are not observed in vehicle-treated animals (data not shown).

In a sub-chronic GLP toxicology study in dogs wherein Compound X was administered orally at 3, 10, and 30 mg/kg/day for 4 weeks histopathology was similar to the shorter-duration study, however hyperplasia was more prominent and significant cholangiocyte necrosis/degeneration was not observed (Figures 1C,D). Additionally, the inflammatory infiltrate was predominantly lymphocytes and plasma cells with rare neutrophils, and a fine extracellular matrix (fibrosis) was observed surrounding affected ducts (Supplementary Figure S2B). At the end of a 4-week recovery period in dogs administered 30 mg/kg/day, the bile duct finding persisted, as did the fibrosis, consistent with an irreversible toxicity (Supplementary Figure S2C). Also concerning was the observation that fibrosis was extending between portal tracts (“bridging fibrosis”). A 2-week toxicology study was conducted in dogs prior to the 4-week, with similar bile duct findings at 30 mg/kg/day (data not shown). As the findings were consistent and qualitatively similar between all three studies, and studies of additional duration were not performed, the precise time of their development following the initiation of dosing is unknown.

Intriguingly, evaluation of clinical pathology in the 5-day, 2-week, and 4-week studies revealed either no or only modest increases in liver enzymes alkaline phosphatase and alanine aminotransferase in individual animals, biomarkers commonly associated with drug-induced liver injury (DILI) (Table 1). This is consistent with the overall lack of hepatocellular pathology in either study. Further consistent with a lack of effect on small bile ductules or hepatocellular canaliculi, there were no increases in bilirubin nor any evidence of cholestasis (canalicular plugging, icterus). Though a similar pathologic finding was evident at 3 and 10 mg/kg/day in the 4-week study, there were no clinical pathology findings at those doses, consistent with a poorly monitorable toxicity.

**TABLE 1:** Clinical pathology data from *in vivo* toxicology studies.

Study	Dose level (mg/kg/day)	Liver enzymes
Compound X × 4 weeks	30	↑ALT (2.4×)*, ↑ALKP (3×)**
Compound X × 2 weeks	30	No significant findings
Compound X × 5 days	30	↑ALKP (2×, 1 of 2)
Compound Y × 5 days	50	No significant findings
Compound Z × 5 days	50	No significant findings

*Elevation from individual baseline mean in a single animal out of 10. **Elevations from individual baseline means in 3 of 10 animals. No liver enzyme increases were observed at 3 or 10 mg/kg/day.

In 2-week and 4-week toxicology studies of Compound X performed in mice, there were no notable test item-related findings in the biliary tree (data not shown).

### Exploratory toxicology studies in dogs with related heterobifunctional degraders did not result in bile duct toxicity

Compounds Y and Z are close analogs of Compound X (Table 2), however with a less basic linker (measured pKa 6.4 for Y and Z vs. 9.0 for X). Compound Z additionally has a highly similar target-binding ligand. In a 5-day exploratory toxicology study in dogs at 50 mg/kg/day with both of these analogs, there were no significant findings in the bile ducts (Supplementary Figures S3A,B) and no clinical pathology changes (Table 1). Unlike Compound X, Compound Z resulted in moderate phospholipidosis in the lung in the dog study (Supplementary Figures S4A,B), characterized by mixed inflammation associated with foamy macrophages.

**TABLE 2:** Chemical structures and physicochemical properties of degraders.

Compound	Compound X	Compound Y	Compound Z
Tanimoto similarity to Compound X	1	0.47	0.62
Target degradation, TMD8 cell, DC50 (µM), 6 h/dMax	0.0007/100%	0.0007/100%	0.0006/100%
Target-dependent cell line, IC50 (µM), 72 h	0.0003	0.0007	0.0005
Primary cholangiocyte viability, IC50 (µM), 48 h	3.4	>100	>100
Molecular weight	783	829	799
Experimental pKa, most basic	9.0	6.4	6.4
cLogP	7.09	6.16	6.89
PAMPA (10E-6 cm/s)	0.125	0.481	0.0072
EPSA	124	115	116
Caco 2, P A-to-B (10E-6 cm/s)/efflux ratio	<0.3/>1.6	3.4/2.8	4.6/2.9
Dog PK, Vss (L/kg)	6.70	No data	2.52

### Disposition of degrader compounds to bile *in vivo*

As bile is the matrix most closely related to cholangiocyte biology, bile was collected from the gall bladder upon euthanasia of study dogs to determine drug level. This analysis for all three compounds revealed high concentrations of administered degrader, from 179 to 396 μg/mL (Table 3), representing bile concentrations 99–194-fold higher relative to terminal plasma concentrations. Liver drug tissue concentrations were variable, with liver: plasma ratios ranging from 17 to 736. High disposition of Compound X is predicted by higher volume of distribution (Table 2) and tissue accumulation has been described with protein degraders (Volak et al., 2023).

**TABLE 3:** Degrader drug level *in vivo* and cholangiocyte cytotoxicity *in vitro*.

Study	Dose level (mg/kg/day)	Terminal plasma drug level (μg/mL)	Terminal bile drug level (μg/mL)	Terminal liver drug level (μg/g)	Cholangiocyte IC50 (μM)
Compound X × 4 weeks	30	0.96	ND	ND	3.9
Compound X × 2 weeks	30	1.93	ND	1,420	
Compound X × 5 days	30	1.81	179	526	
Compound Y	50	2.04	396	70.4	>100
Compound Z	50	1.99	197	33.9	>100

### Distribution of degrader compounds to cholangiocytes *in vivo*

**FIGURE 2:**
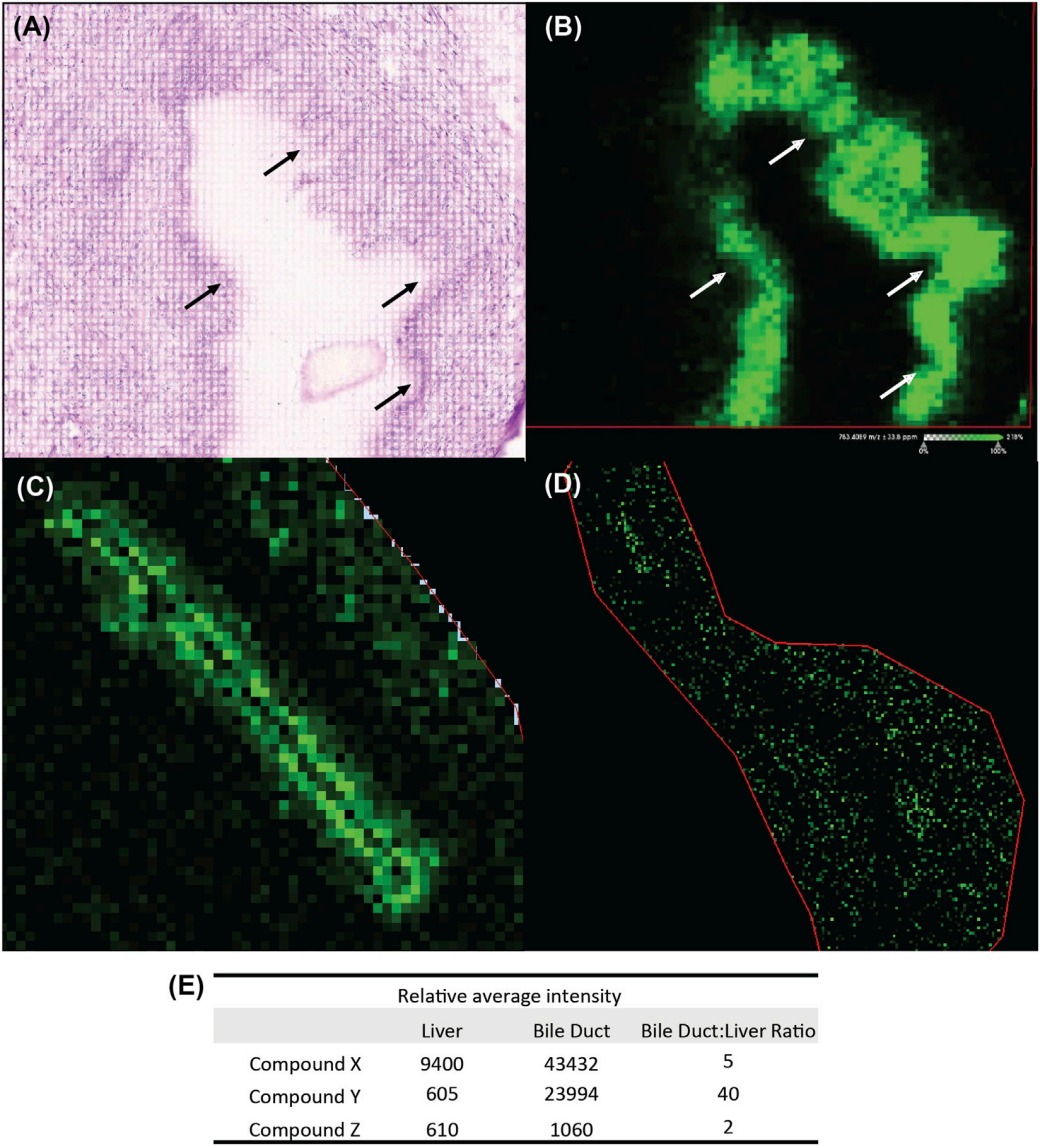
Administration of Compound X results in distribution to cholangiocytes as detected by mass spectrometry (MS) imaging. **(A)** Histology of frozen liver section showing region of interest to include a large bile duct from a dog administered Compound X for 28 days. **(B)** Corresponding MS image showing signal enrichment in bile duct (arrows) as compared to surrounding hepatocellular parenchyma. **(C)** Compound Y administered to dogs for 5 days is also enriched in bile duct as compared to surrounding tissue. **(D)** Compound Z has overall low abundance in liver tissue. **(E)** Pixel quantification as average intensity for all three compounds in bile duct and surrounding liver tissue.

Mass spectrometry imaging (MSI) can be deployed as a supplement to tissue drug concentrations by applying a spatial context to drug distribution within a tissue (Prideaux and Stoeckli, 2012; Lamont et al., 2021). Frozen liver samples for MSI revealed high signal intensity of degrader concentrated in large bile ducts (Figures 2A–C) for Compound X and Compound Y. This finding indicates preferential distribution in tissue to the bile duct. The signal was predominantly of the parent molecule, indicating a lack of significant metabolite generation. Further, the ratio of signal intensity between the bile duct and the liver ranged from 2 to 40 (Figure 2E). For Compound Z, overall signal in the liver and bile ducts was low (Figure 2D), consistent with the lower drug level in liver overall (Table 3).

### Compound X induces target and CRBN-independent cytotoxicity in human cholangiocytes

**FIGURE 3:**
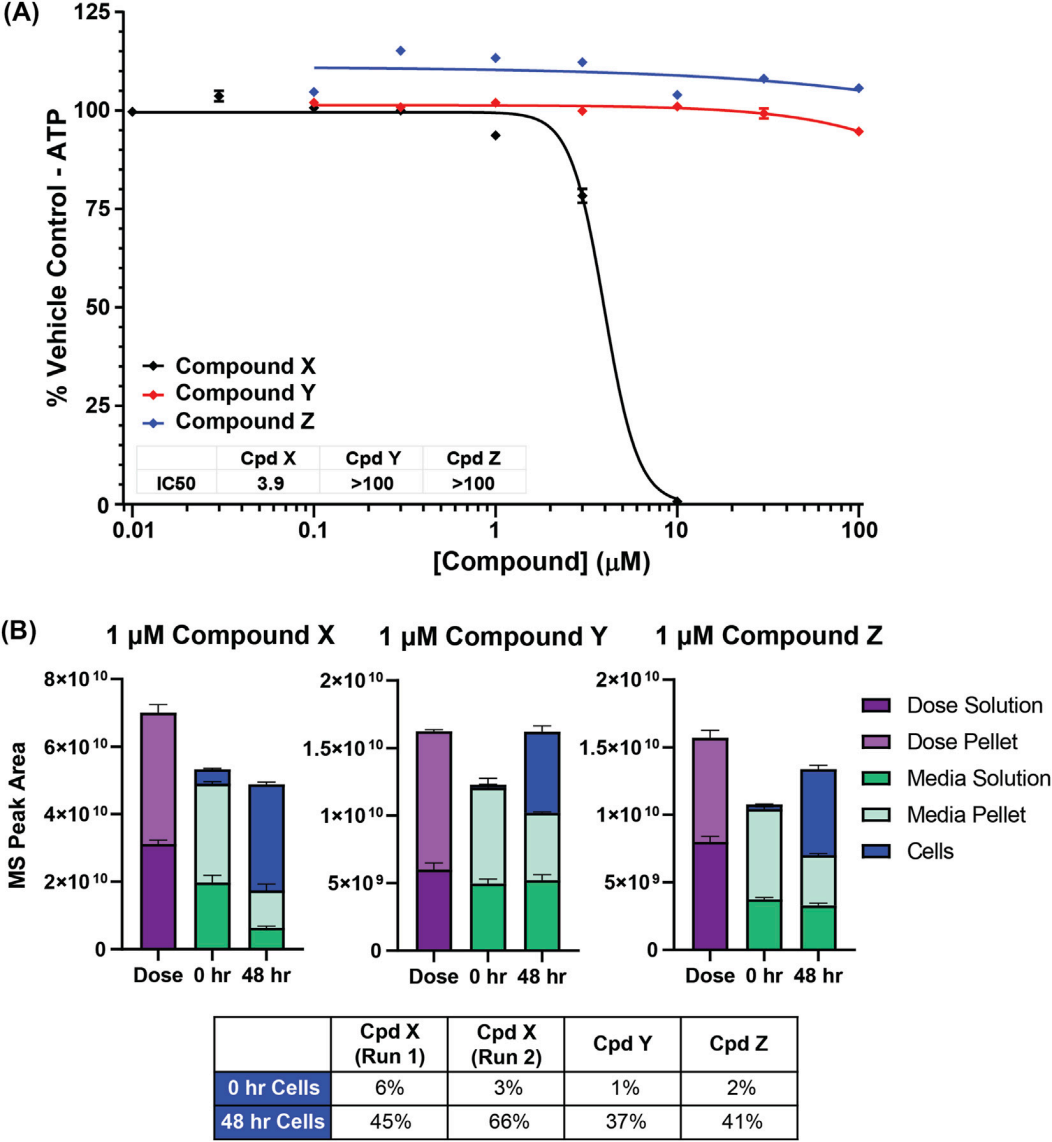
*In vitro* data with primary human cholangiocytes. **(A)** Compound X but not Compound Y nor Compound Z is cytotoxic to cholangiocytes at low µM concentrations. **(B)** All three degraders are taken up into cholangiocytes as detected by targeted LC-MS analysis.

In order to investigate the potential mechanism of the observed toxicology findings in bile ducts, primary human cholangiocytes were obtained. Titration of Compound X on these cells induces cytotoxicity in a time- and dose-dependent manner, resulting in an IC50 of 3.9 μM (repeated in numerous independent experiments over 3 years) in 48 h (Figure 3A). In contrast, less basic analog degraders Compound Y and Compound Z are not toxic to cholangiocytes at concentrations up to 100 μM (Figure 3A).

Of note, the target-dependent cellular IC50 of Compound X in the relevant cell line for efficacy evaluation is 0.0003 µM (Table 2), and target expression in human cholangiocytes is below the limit of detection (Lonsd et al., 2013). Similarly, target transcripts are not detectable in dog tissue (data not shown). A methylated-inactive version (Lu et al., 2015) of Compound X (Compound A) that has a significantly reduced binding activity to cereblon E3 ligase (CRBN) (target degradation DC50 0.49 µM as compared to 0.0007 µM for Compound X) has an overlapping IC50 with Compound X in cholangiocytes (Supplementary Figure S5A). Additionally, siRNA-mediated knockdown of CRBN in human cholangiocytes did not alter the IC50 of Compound X in these cells (Supplementary Figures S5B,C). Altogether, these data suggest that neither target nor the CRBN-mediated degradation activity of Compound X are relevant to the observed cytotoxicity in cholangiocytes.

### Compounds X, Y, and Z are significantly less cytotoxic to human hepatocytes

Consistent with histopathology data demonstrating lack of toxicity in hepatocellular parenchyma, Compound X is significantly less toxic to primary human hepatocytes (IC50 > 30 μM in a cholestatic hepatotoxicity assay [C-DILI], Supplementary Figure S6). Therefore, human cholangiocytes are >10-fold more sensitive to Compound X as compared to human hepatocytes *in vitro*. The IC50 of Compound Y and Compound Z for hepatocytes is >100 μM and these compounds were considered to have no cholestatic potential in the assay (Supplementary Figure S6).

### Intracellular accumulation of degrader compounds in cholangiocytes

Due to insufficient resolution of MSI to confirm distribution of degrader compounds at a cellular level for tissue samples, we instead used liquid chromatography tandem mass spectrometry (LC-MS) to quantify the uptake of Compound X into primary human cholangiocytes. After 48 h in culture, 45%–66% of administered Compound X was present in the cellular fraction in two independent experiments (Figure 3B). Comparable degrees of cellular uptake of Compound Y and Compound Z were observed (37% and 41%, respectively, Figure 3B), suggesting that degrader compound uptake into cholangiocytes may be necessary but not sufficient for cytotoxicity.

### Proteomics analysis in cholangiocytes reveals Compound X-induced defects in intracellular protein transport

Off-target effects, and in particular off-target degradation, are key safety concerns in the development of heterobifunctional degraders. Our data suggest that CRBN recruitment and target degradation by the ubiquitin-proteasome system are not significant factors in Compound X-mediated cytotoxicity in cholangiocytes. For further validation, and to identify alternative hypotheses for the mechanism of toxicity, we performed unbiased proteomics studies in primary human cholangiocytes sourced from three vendors and treated with Compound X (Supplementary Figure S7A). As expected, the target was not detected in the 6,000–8,000 proteins quantified in each study. Additionally, as the intended target is a kinase, we evaluated other detected kinases (>180 kinases detected in each study), and found none were detected with significant reductions in protein abundance that could be attributed to degradation.

In the absence of a clear degraded off-target, we performed a pathway enrichment analysis of proteins with significant changes in abundance following Compound X treatment (Zhou et al., 2019). Surprisingly, we found the top two enriched pathways were populated by independent sets of genes that largely had *increases* in protein abundance (Supplementary Figure S7B). One set comprised proteins involved in intracellular protein transport to cellular vacuoles, which was of particular interest given an increased number of autophagosomes seen by EM in the cholangiocytes of dogs following dosing with Compound X (Figures 1F,G). The other contained a set of secreted proteins that are phosphorylated in the Golgi or endoplasmic reticulum (see Supplementary Note).

### Compound X treatment is followed by LC3B lipidation and increased autophagosomes

**FIGURE 4:**
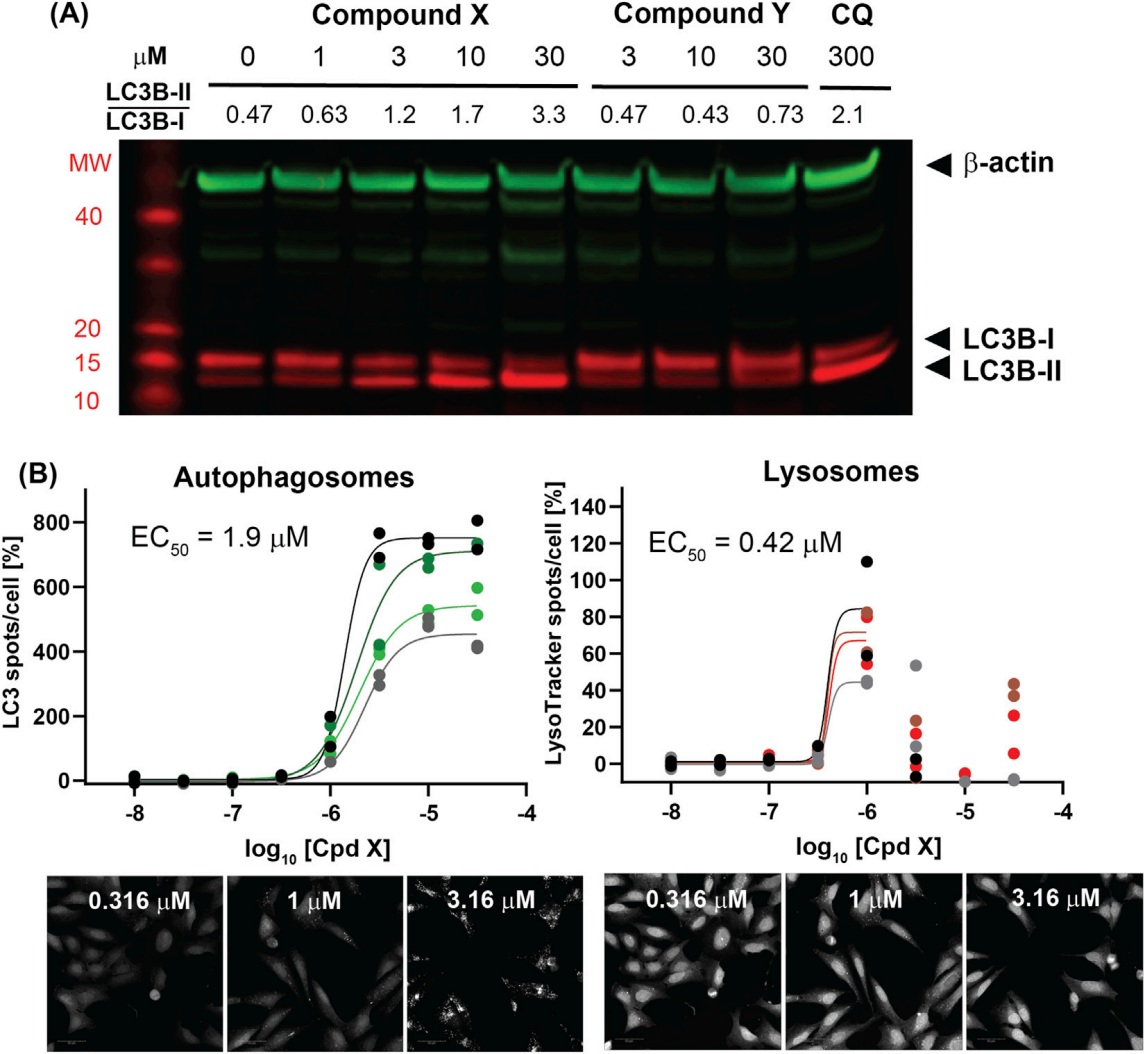
**(A)** Compound X but not Compound Y induces an increase in LC3B lipidation in primary cholangiocytes after 3 h treatment. **(B)** A dramatic increase in autophagosomes is observed by high-content imaging of U-2 OS GFP-LC3B cells following Compound X treatment for 3 h. A modest increase in lysosome number precedes a reduction in LysoTracker signal. % Activation denotes the number of GFP-LC3B or LysoTracker spots relative to values from the same plate for a positive/high control (3 µM Torin-1), set to 100%, and the vehicle control, set to 0%. EC50 values displayed represent the geometric mean of values from the individual replicates shown; concentrations with reduced LysoTracker values at >1 μM were excluded from fits.

One protein involved in intracellular protein transport that was identified as significantly increased following treatment with Compound X but not Compound Y is p62 (Supplementary Figures S7C,D), suggesting a defect in lysosomal degradation. p62 can serve as a surrogate marker for inhibition or dysfunction of autophagy (Liebl et al., 2022), but as it only modestly increased after an 18 h incubation of near-cytotoxic concentrations of Compound X (IC50 of 3.9 μM), we decided to investigate another commonly used autophagy marker, LC3B. LC3B is lipidated and localizes to autophagosomes upon autophagy activation; while total LC3B abundance changes can be minimal, an increase ratio of the lipidated (LC3B-II) to the non-lipidated (LC3B-I) forms can indicate an inhibition at a later autophagy stage, such as autophagosome-lysosome fusion. Upon just a 3-h incubation in cholangiocytes, selected to minimize the cytotoxic effects of Compound X, we observed a concentration-dependent increase in the LC3B-II/LC3B-I ratio (Figure 4A). This effect is similar to that induced by chloroquine (CQ), a well-studied late-stage autophagy inhibitor. No change of LC3B lipidation was observed with Compound Y treatment. Following this *in vitro* observation, an LC3B IHC stain was applied to liver tissue from dogs administered Compound X and demonstrated increased positive staining in hyperplastic cholangiocytes as compared to vehicle control dog tissue (Supplementary Figure S8).

As immunoblot-based LC3B assays are only semi-quantitative, we next tested Compound X in a high-content imaging assay designed to quantify autophagosomes and lysosomes and able to discriminate between autophagic flux activation and inhibition. This assay utilizes U-2 OS, an osteosarcoma cell line that is not dependent on the target (Tsherniak et al., 2017) and has a similar sensitivity to Compound X as cholangiocytes (Supplementary Figures S9A,B). Imaging and analysis of the U-2 OS GFP-LC3B reporter cell line (see Supplementary Figure S10A for schematic) showed a dramatic increase of autophagosome number (LC3 spots per cell, EC50 = 1.9 μM) following a 3-h incubation with Compound X, over 5-fold higher than the assay’s positive control, the autophagy activator Torin-1 (Figure 4B). A near-identical increase was seen by Compound A (methylated inactive compound, EC50 = 1.4 μM, Supplementary Figure S10B), suggesting that CRBN recruitment by Compound X is not related to its disruption of autophagy. A minimal change in autophagosome number was seen by either Compound Y or Compound Z at evaluated concentrations (Supplementary Figures S10C,D). In evaluating lysosome number by LysoTracker puncta, Compound X induced a modest increase up to 1 μM; while at higher Compound X concentrations, the LysoTracker signal precipitously dropped, suggesting either Compound X increases the lysosomal pH by blocking acidification, or accumulates in lysosomes, blocking LysoTracker lysosomal trapping, or both (Kazmi et al., 2013) (Figure 4B).

While this quantitative high-content imaging assay in U-2 OS cells and the changes to p62 and LC3B in human primary cholangiocytes in culture generally recapitulates the more qualitative microscopic observations of increased autophagosomes/lysosomes within the dog bile ducts, it is unclear and in fact improbable that these *in vitro* models exactly replicate the quantitative molecular changes which occurred *in vivo* (e.g., relative increases in lysosomes vs. autophagosomes). Indeed, the cholangiocytes in each are from different species and exist in dramatically different contexts of cholangiocytes in each: a two versus three-dimensional organization and, importantly, direct exposure to media *in vitro* and to bile *in vivo*.

### CRISPR-Cas9 screening reveals the lysosomal V-ATPase is required for LC3B lipidation and Compound X cytotoxicity

While the above data show Compound X is associated with late-stage autophagy inhibition and a potential increase of lysosomal pH, they do not causally link these effects to its cytotoxicity in cholangiocytes. We therefore performed two separate whole genome CRISPR screens in engineered U-2 OS lines: the first expressing Cas9 to identify candidate genes conferring sensitivity or resistance to Compound X when knocked out (CRISPR KO (Doench et al., 2016)); the second, with a catalytically dead Cas9 gene fused to a transcriptional activator, used to identify genes that confer sensitivity or resistance to Compound X when overexpressed (CRISPR activation, or CRISPRa (Sanson et al., 2018)).

**FIGURE 5:**
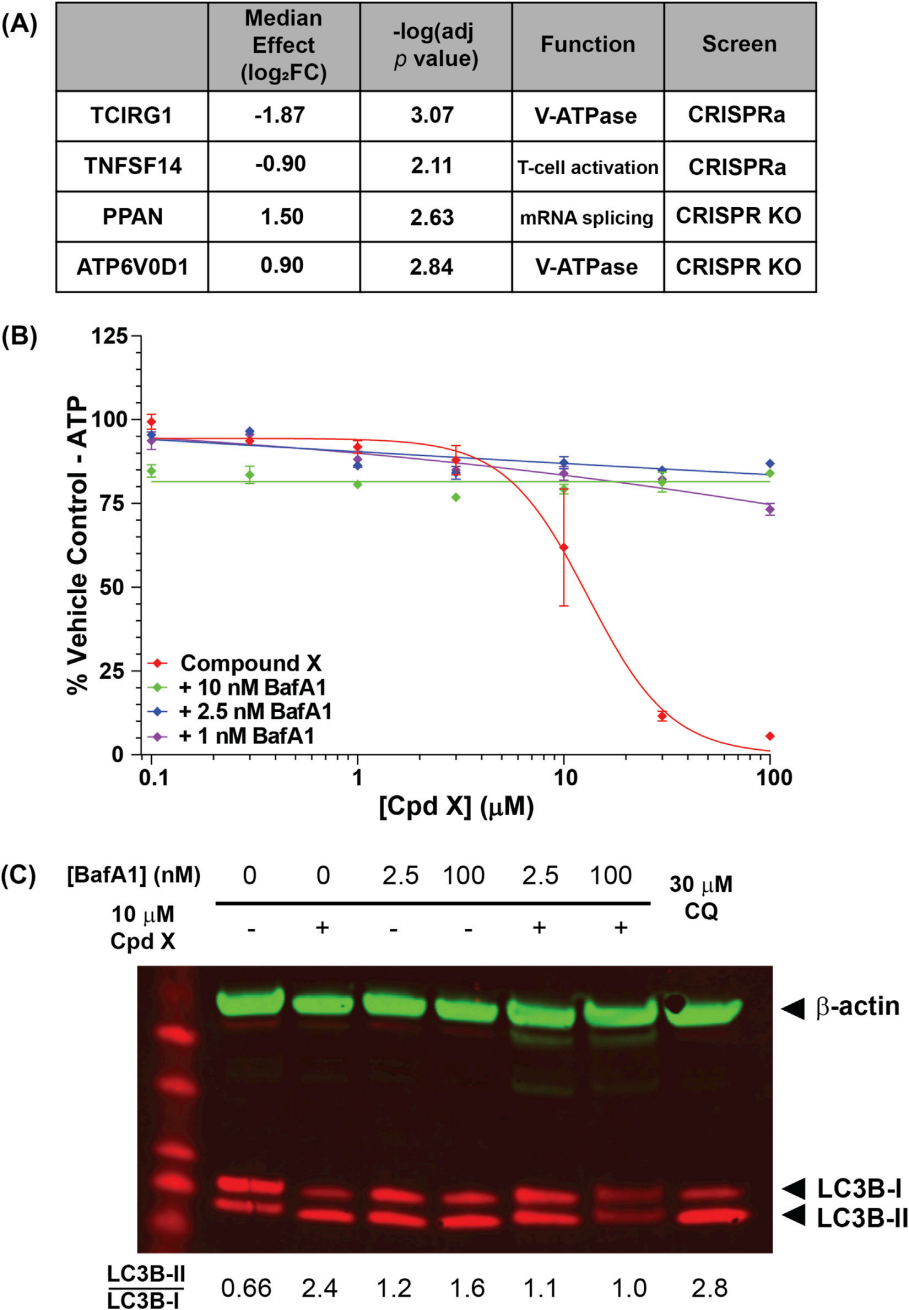
**(A)** U-2 OS genes that are significantly enriched or depleted upon knockout or overexpression following Compound X treatment. Two hits are subunits of the lysosomal V-ATPase subunit. **(B)** The V-ATPase inhibitor bafilomycin A1 (BafA1) restores viability of cells incubated for 6 h with Compound X. **(C)** BafA1 additionally reduces lipidation of LC3B in primary cholangiocytes following Compound X treatment.

Consolidating results from multiple guides to individual genes revealed four gene “hits” that were significantly enriched or depleted (adjusted *p* value <0.05) in the two screens (Figure 5A; Supplementary Figures S11A,B). Strikingly, two of the four were subunits of the vacuolar-type ATPase (V-ATPase): upon Compound X treatment, ATP6V0D1 KO cells were enriched, while TCIRG1 (T-cell immune regulator 1) overexpression cells were depleted, suggesting that the V-ATPase as a whole is a key requirement of Compound X cytotoxicity.

The V-ATPase is a ubiquitously expressed complex that localizes to late endosomes and lysosomes, performing the compartment acidification required for lysosomal degradation (Toei et al., 2010). To confirm that V-ATPase function mediates Compound X cytotoxicity in not just U-2 OS cells, but also in primary human cholangiocytes, we co-treated cells with Compound X and the V-ATPase inhibitor bafilomycin (BafA1) and found that the latter fully rescued compound-induced cytotoxicity at <24 h (Figure 5B), whereas BafA1 alone was cytotoxic at longer timepoints (data not shown). Co-treatment of Compound X with a saturating (100 nM) or sub-saturating (2.5 nM) concentration of BafA1 (Liebl et al., 2022) for 3 h also reduced the LC3B lipidation by Compound X (Figure 5C), demonstrating a temporary rescue of autophagy defects induced by Compound X.

Together, these data causally link the cytotoxicity of Compound X in primary cholangiocyte culture to disruptions in intracellular transport via autophagy and lysosomal degradation. Further, autophagosomes were observed in cholangiocytes *in vivo* (Figures 1F,G; Supplementary Figure S8), associated with cytotoxicity and subsequently bile duct hyperplasia in dogs. Importantly, these *in vitro* and *in vivo* effects did not occur following exposure to analog degraders with less basic linkers, despite unaltered distribution to cholangiocytes, suggesting that this mechanism of toxicity is driven by physicochemical properties of the degraders.

## Discussion

A systematic review of preclinical translatability for DILI concluded that a mechanistic investigation is more successful in predicting clinical safety, as compared to *in vivo* or *in vitro* toxicology assessments alone (Dirven et al., 2021). In this study, the mechanistic drivers for a significant toxicology finding in dogs, bile duct hyperplasia with secondary inflammation and fibrosis, were investigated and identified. The physicochemical properties (notably lipophilicity and linker basicity) of a novel degrader compound were shown to drive cholangiocyte injury *in vitro* and *in vivo*. Compound X, but not the less basic analogs evaluated, resulted in late-stage autophagy inhibition and cytotoxicity in primary human cholangiocytes, translating to bile duct injury with increased LC3B-labeled autophagosomes in the dog. This is despite the lack of significant difference in uptake or distribution to cholangiocytes *in vitro* and *in vivo* between these basic and less basic compounds, suggesting that drug accumulation in cholangiocytes is necessary, but not sufficient, to drive toxicity. This is further demonstrated by the observation that Compound Y has a higher terminal bile drug level as compared to Compound X but lacks hepatobiliary toxicity.

The mechanism of cholangiocyte uptake of these large degrader compounds is unknown. After 48 h of treatment, over 37% of Compound X administered to cells were taken up, higher than would be predicted based on the compound solubility in media, or its permeability, suggesting active uptake of compound (soluble or in aggregates) by cholangiocytes. Indeed, receptor-mediated endocytosis is known to occur in cholangiocytes as one of their primary functions is to “sample” the bile as a defense mechanism against xenobiotics (Xia et al., 2006; Ishii et al., 1990; Tabibian et al., 2013). This hypothesis is also supported by our observation of increased serum proteins within primary cholangiocytes after treatment with Compound X (see Supplementary Note). A recent study identified the interferon-induced transmembrane proteins (IFITMs) as mediating active uptake of large, bifunctional compounds in K562 lymphoblast cells (Lou et al., 2022). As knockout or overexpression of IFITMs in our CRISPR screens did not alter the sensitivity of U-2 OS cells to Compound X, it may be possible that uptake mechanisms are compound and even cell lineage-dependent.

While it is documented that there is functional heterogeneity between cholangiocytes in small and large bile ducts (Kanno et al., 2000), it is unclear why the toxicity we observed in dogs was limited to large bile ducts. Large but not small bile ducts are actively involved in bile modification including resorption of bile acids, amino acids, and glucose with secretion of electrolytes and water (Kanno et al., 2000; Trauner and Boyer, 2003; Fukushima et al., 2006). Perhaps if the accumulation of degraders within large cholangiocytes is selectively mediated by active uptake by these cells, large bile ducts would be more susceptible. Other potential contributors to this observed sensitivity *in vivo* may be changes in bile pH and composition within large ducts as opposed to bile ductules. There are few drugs which are known to target large ducts as a result of intrinsic toxicity, leading to a histologic picture that resembles primary sclerosing cholangitis in humans, an irreversible scarring disease causing stenosis of large bile ducts (Pollheimer et al., 2011). Similarly to humans, toxicologic impacts on the liver in animals are more frequently observed in hepatocytes and in small bile ductules. No examples of large bile duct injury or toxicity limited to the large bile duct as described here in animals were found in the literature.

Physicochemical properties of high (basic) acid dissociation constant (pKa) and high lipophilicity (ClogP) are characteristics of cationic amphiphilic drugs (CADs), such as chloroquine (CQ), which have been documented to cause drug-induced phospholipidosis (PLD) (Ploemen et al., 2004). While PLD was not a significant feature of Compound X-induced toxicity at doses evaluated in dogs, it was observed in the 5-day study with Compound Z and is predicted as a risk with all three compounds *in vitro* (Supplementary Figure S4C). Based on these data and importantly the lack of colocalization of PLD and biliary toxicity, we suggest that while similar physicochemical properties may drive both, they are not mechanistically linked. Further supporting this conclusion, PLD was observed in toxicology studies in mice with Compound X in the absence of biliary toxicity. The reason for species-sensitivity in the occurrence of bile duct toxicity is not known but may relate to biliary composition and metabolism differences between rodents and dogs (Zheng et al., 2024).

The increase in autophagosomes in the dog large bile duct (as seen ultrastructurally and by LC3B IHC), cultured human cholangiocytes (by LC3B Western blot), and in the U-2 OS autophagy assay following dosing with Compound X is a key molecular marker of this toxicity and points to late-stage inhibition of autophagy. A precipitous drop in LysoTracker signal observed with Compound X but not with less basic analogs suggests that the basicity is leading to accumulation within the lysosome. Lysosomal trapping within the cytosol of these cells may also contribute to the high intracellular concentration and retention of the compound. Inhibition of the V-ATPase by BafA1 may raise the lysosomal pH sufficiently to reduce lysosomal trapping of Compound X and therefore alleviate cytotoxicity. The V-ATPase has also been shown to mediate conjugation of ATG8s to endolysosomal single membranes (CASM) caused by lysomotrophic compounds in a process that is similar to LC3-associated phagocytosis and entosis (Florey et al., 2015; Jacquin et al., 2017). Mechanistically, the V-ATPase acts as a convergence point in these processes by recruiting ATG16L1 and thereby the downstream lipidation machinery (Hooper et al., 2022). Our data suggest that inhibiting V-ATPase with BafA1 or eliminating a key subunit that prevents V0-V1 binding can partially alleviate excess LC3B lipidation by disrupting V-ATPase’s function during CASM.

While we have highlighted similarities in the cellular effects of Compound X and CADs such CQ, there is a key difference: the target tissue of the large bile duct. While Compound X is, like CADs, basic and lipophilic, it does also belong to a distinct chemical space–larger and even more lipophilic (Poongavanam and Kihlberg, 2021; Volak et al., 2023). In addition to differences in their physicochemical properties, the tissue specificity of the Compound X toxicity can be partially explained by its high accumulation in bile and bile excretion, in contrast to renal clearance for CQ (Müller et al., 2011). High tissue accumulation and retention within tissue by some degraders has been previously reported (Zhang et al., 2024); it is possible that specific and distinct physicochemical-associated toxicities may occur, depending on the distribution across tissues and excretion route, as well as the compound’s specific molecular features.

Importantly, the discovery of close analogs of Compound X lacking the liver findings while maintaining desired efficacy and absorption, distribution, metabolism, and excretion (ADME) characteristics demonstrate that these physicochemical and toxicity challenges are not universal to bifunctional degraders, nor an insurmountable challenge to this specific chemical series. *In vitro* assays described herein (i.e., cholangiocyte cytotoxicity and autophagy assays) have been used to prioritize degrader compounds for *in vivo* dog studies, thereby reducing overall animal usage and accelerating development of safer therapeutics.

## Data Availability

The mass spectrometry proteomics data have been deposited to the ProteomeXchange Consortium via the PRIDE partner repository with the dataset identifier PXD071598.
