# Supplementary material for: Off-target autophagy disruption associated with a novel liver toxicity in dogs for a highly basic heterobifunctional protein degrader
Source: Front Pharmacol. 2025 Dec 10;16:1664889. doi: 10.3389/fphar.2025.1664889 (PMC12727973; doi:10.3389/fphar.2025.1664889)
Supplement: Supplementary file 1 [file DataSheet1.docx]

**SUPPLEMENTARY MATERIAL**

**Supplementary Experimental Procedures**

*Mass Spectrometry Imaging*

Livers were sectioned on a Thermo HM550 cryostat at -16°C. The section thickness was set at 10 µm. The slides were dried and stored in a vacuum desiccator for not more than 1 week before acquisition. Matrix coating was applied using a HTX TM sprayer M3+. For Compound Y and Compound Z, the coating was applied by automatic spraying of CHCA solution (75 mg CHCA, 7.5mL ACN, 2.5 mL H_2_O, 200 µL TFA): 8 passes, 0.1 mL/min of flow rate, 60°C nozzle temperature, 2 mm/track, 1100 mm/min of nozzle moving speed, 40 mm of nozzle height, 10 PSI nitrogen pressure, and Criss Cross spraying mode. For Compound X, the coating was applied with spraying of DHA solution (100 mg DHA, 9 mL ACN, 1 mL H_2_O, 150 µL TFA): 4 passes, 0.1 mL/min of flow rate, 1100 mm/min of nozzle moving speed, 2.0 mm/track, 40 mm of nozzle height, 70°C of nozzle temperature, 10 PSI nitrogen pressure, and Criss Cross spraying mode. MSI sessions of matrix coated sections were carried out on a Bruker timsTOF fleX mass spectrometer. In general, the laser was set for 50-100 shots/pixel with laser frequency at 10,000 Hz. Stage and smart beam profile (spatial resolution) were varied from 5-10 µm, with trapped ion mobility active. Positive ion mode was used. MALDI-2 was used for Compound Y and Compound Z with laser frequency at 1000 Hz. Compound X was imaged with MALDI-1 only. Data was processed with Bruker SCiLs Lab MVS 2024a Pro.

*Sample Preparation and LC-MS Analysis of Cholangiocyte Fractions to Determine Compound Abundance*

Fractions were processed by addition of organic quench solution (9:1 v/v acetonitrile/methanol with 1 nM verapamil added as an internal standard). For cholangiocyte pellets, 1 mL of quench solution was added twice to each well, followed by scraping, and the resulting 2 mL combined solution was clarified at 2800*g* for sample isolation. For input media, spent media, and wash fractions, 100 mL of sample was combined with 600 mL organic quench solution. Before LC-MS analysis, all samples were diluted with an equivalent volume of milli-Q water to yield an approximately 50/50 (v/v) organic/aqueous sample composition for HPLC compatibility.

LC-MS analysis was performed on a on Shimadzu Prominence UPLC and ABSciex 6500 QTrap Triple Quadrupole mass spectrometer system. 5 mL of each sample was injected onto a Waters CORTECS T3, 2.7 μm, 3.0 × 50 mm column and separated using 0.1% formic acid (FA) in water as mobile phase A and 95% acetonitrile in water with 0.005% FA as mobile phase B. After a 0.5 min hold at 5% mobile phase B, compounds were eluted over a 1 min gradient up to 95% B, followed by a 1 min hold at 95% B and re-equilibration at 5% B for 0.5 min. MS data were acquired in multiple reaction monitoring (MRM) mode using optimized collision energies, entrance potentials, and delustering potentials for each analyte. The MRM transitions used for quantification were, m/z 783.5 → 315.2 for Compound X, m/z 799.4 → 630.3 for Compound Y, 829.3 → 660.3 for Compound Z. Analyte peak areas were calculated with the IntelliQuan algorithm in Analyst 1.6.2 software and mass balance was calculated in Microsoft Excel.

*Cholangiocyte and Hepatocyte Cell Culture, CRBN knockdown, and Viability Testing*

For the C-DILI cholestatic hepatotoxicity assay, cryopreserved primary human hepatocytes were suspended in BioIVT QUALGRO Seeding Medium (BioIVT, Westbury NY USA) at a density of 0.8 million viable cells/mL and plated at 70 μL per well onto collagen-coated BioCoat 96-well cell culture plates pre-filled with 40 μL BioIVT QUALGRO Seeding Medium. 24 hours later seeding media was removed and cells are overlaid with 125 μL BioIVT culture medium supplemented with 0.25 mg/mL Matrigel. Cells were maintained for 2 days. On Day 4, dosing solutions were prepared by diluting test article and control stock solutions into QUALGRO C-DILI Culture and QUALGRO C-DILI Sensitization media (BioIVT, 0.125 ml/well). 0.1% DMSO was used as the vehicle control, Troglitazone (75 μM) was used as a positive cholestatic control. All treatments were prepared in triplicate. Following 24 hours of drug exposure, cell culture medium was harvested for LDH enzyme leakage assessment (CytoTox-ONE, Cat No. G7890, Promega, Madison WI USA) and ATP content determination was run on the cell lysate (CellTiter-Glo, Cat No. G7570, Promega).

For cholangiocyte viability following knockdown of CRBN, cells were seeded at 5,000/well on collagen-coated white clear bottom 96-well plates (Corning #256701) and allowed to attach for ~4h. At 4h and 24h post-attachment, cells were transfected with 3 μM concentrations of siRNA targeting CRBN (Horizon Discovery Cat#E-021086-00-0050) and a Non-Target control (Horizon Discovery Cat#D-001910-10-50), respectively. Additionally, a siRNA untreated control was included. Following the second siRNA treatment at 24h, Compound X compound was dispersed in triplicate at half-log concentrations (0.1-30 μM) using the TECAN compound dispenser, normalizing dimethyl sulfoxide (DMSO) vehicle content to 0.1% for all wells. Media was aspirated and CellTiter Glo 2.0 was added to determine cytotoxicity. Plates were shaken for ~2 min and allowed to sit for a minimum of 10 min for signal stabilization. Luminescence was determined using the Clariostar (BMG Labtech, Ortenberg Germany). Data were fit using GraphPad Prism, utilizing the nonlinear regression model log(inhibitor) vs response – variable slope (four parameters), with the bottom parameter constrained to zero.

*Phospholipidosis analysis in HepG2 cells*

HepG2 cells (ATCC, HB-8065), maintained in phenol red free MEM (Gibco 51200-038) supplemented with 10% FBS (Gibco 16000044), 1x sodium pyruvate (Gibco 11360070), and 1X pen/strep (Gibco 15140122) were utilized for PLD assessment by high content imaging, using the HCS LipidTOX Phospholipidosis and Steatosis Detection Kit (Invitrogen H34158), according to kit protocol, with Hoechst 33342 Dye (Thermo 62249) added for nuclei staining. For assay, cells were seeded at 10,000/well on collagen coated 96W black plates (Corning 354649) and allowed to attach for ~4h. Following cell attachment, media was exchanged with 50 μl media and 50 μl of 2X media/dye made from a 500X dilution of LipidTOX™ Red phospholipidosis detection reagent, then compound was dispersed in triplicate at half-log concentrations (0.03-30 μM) using the TECAN compound dispenser, normalizing DMSO content to 0.1% for all wells. Propanolol at 30 μM was included as a positive control. Plates were incubated for 24h and dosed again after media exchange as described above for an additional 24h. In preparation for image acquisition, an 8% solution of paraformaldehyde (16% stock EM grade, Electron Microscopy Sciences 15710-S) in PBS containing 10,000-fold dilution of Hoechst dye was added directly to the plate. After 30 min incubation at room temperature, fixative solution was removed and plate washed 3 times with PBS, with a final addition of PBS for imaging. Using a Biotek Cytation 5 cell imaging reader, nuclei and sum intensity were capture using the DAPI and Texas Red filter sets. Sum intensity data was normalized to cell count and plotted using GraphPad Prism.

*Sample Preparation for Proteomics*

Sample proteomes from Compound X or vehicle-treated cholangiocyte pellets were prepared for LC-MS/MS analysis using the Preomics iST 96x (P.O.00027, for ScienCell cholangiocytes treated in triplicate with 0, 0.3, 1, and 3 µM Compound X) or iST-NHS 96x kit (P.O.00030, for ABM and Creative Bioarray cholangiocytes, treated in duplicate with 0, 1, and 10 µM Compound X). In brief, cell pellets from cholangiocytes treated with Compound X or vehicle were resuspended in 50-100 mL Preomics LYSE buffer from the corresponding kit and lysed within 1.5 mL Bioruptor Plus TPX microtubes (Diagenode C30010010) using a Bioruptor Plus instrument (4 °C, 10 cycles, 30s on, 30 s off).

Lysate protein content was determined with the Pierce 660 nm Protein Assay Kit and normalized as necessary across samples to be compared (first set: ScienCell cholangiocyte samples; second set: ABM and Creative Bioarray cholangiocyte samples). Proteins in lysate were then reduced and denatured for 10 min at 95 °C and digested to tryptic peptides for 1.5h at 37 °C. Peptides for the 12 samples from ABM and Creative Bioarray cholangiocytes (but not ScienCell cholangiocytes) were labeled for 2h with TMTPro isobaric labeling reagents (100 mg per sample, in dry acetonitrile at 30% final concentration), before quenching labeling with hydroxyamine (0.5% final concentration).

Individual samples from either set were then isolated with solid phase extraction following the corresponding Preomics kit protocol. Eluted peptides were pooled into a single sample for TMT-labeled ABM and Creative Bioarray cholangiocyte samples, or kept separate for ScienCell cholangiocyte samples. Peptides were then dried on a Labconco CentriVap benchtop concentrator at 25 °C and resuspended in 20 mL water with 0.1% formic acid (FA) (for ScienCell cholangiocyte samples) or 200 mL water with 0.1% trifluoroacetic acid (for pooled TMT-labeled ABM and Creative Bioarray cholangiocyte samples).

Prior to LC-MS/MS analysis, 400 ng peptide content from each SciencCell cholangiocyte sample was individually loaded onto Solid Phase Extraction (SPE) Evotips (Evosep EV2011). Tips were prepared and washed after sample loading following manufacturer specifications.

The pooled TMT samples, in contrast, were fractionated using a Waters Acquity UPLC by injection onto an Acquity UPLC BEH C18 column (Waters 186002352, 1.8 mm, 100 x 2.1 mm). 20 mM ammonium formate pH 8 (adjusted with 70% ammonia) was used as mobile phase A and 100% acetonitrile (MeCN) as mobile phase B; after 2 minutes at 8% B, peptides were eluted over a linear gradient from 8 to 45% B over 38 min at a flow rate of 0.45 mL/min, followed: by a rapid increase to 90% D over 4 min; a column wash at 90% D for 2 min; a return to 10% D over 2 min; a final reduction to 0% D over 2 min; and finally a 2 min column re-equilibration. Fractions were collected from minute 2 through 42 in a 96-well deep well plate (Waters #186005837), ~180 mL per well along sequential rows of the plate. From this plate, a total of 12 fractions were collected along columns (orthogonal to the direction of sample collection). The pooled fraction samples were then dried in a CentriVap and resuspended in 20 mL water 0.1% FA.

*LC-MS/MS for Proteome Analyses*

Peptides from TMT-labeled, pooled and fractionated peptide samples from ABM and Creative Bioarray cholangiocytes were analyzed using a Orbitrap Fusion Lumos Tribrid mass spectrometer equipped with a EASYnLC 1200 liquid chromatography system (Thermo Scientific), using water 0.1% FA as mobile phase A and 80% MeCN 0.1% FA as mobile phase B (Optima LC-MS grade for both). 2 mL of each sample was injected onto an EasySpray PepMap C18 column (Thermo ES903, 100Å, 2 μm, 50 cm × 75 μm) and eluted over a linear gradient from 6 to 32% B over 166 min at a flow rate of 250 nL/min. The Orbitrap was operated in a data-dependent acquisition (DDA) manner in a positive ion mode using previously reported parameters (Malchow et al. 2022).

Peptides from ScienCell cholangiocyte samples loaded onto Evotips were analyzed using a timsTOF Pro 2 mass spectrometer (Bruker Daltonics) equipped with an EvoSep One liquid chromatography system, using water 0.1% FA as mobile phase A and acetonitrile 0.1% FA as mobile phase B (Optima LC-MS grade for both). Each Evotip-loaded sample was injected onto a ReproSil-Pur C18 column (EvoSep 1106, 1.9 µm, 150 mm x 150 µm) and eluted over the pre-defined, 44 min 30 SPD EvoSep One gradient. Data-independent acquisition (DIA) mass spectrometry of eluted peptides was operated in the parallel accumulation-serial fragmentation (dia-PASEF) mode as previously described^43^. Ramp and accumulation times were set to 120 ms, with dia-PASEF windows of 20 m/z × 0.1 1/K0, spanning 360 to 1200 m/z and 0.7 to 1.3 1/K0.

*LC-MS/MS Data Analysis*

For TMT DDA MS data from ABM and Creative Bioarray cholangiocyte-derived samples, peptides were identified and the relative peptide/protein abundance among samples quantified with MaxQuant (v1.6.15) containing an integrated Andromeda search engine. MS/MS spectra were searched against the human reference proteome (UniProt proteome ID UP000005640, Swiss-Prot entries without isoforms, accessed 4 Jan 2021) with MaxQuant’s build-in list of common contaminants, using the Reporter Ion MS3 parameter for quantification with the TMTPro modification and reporter ions as internal (lysine) and terminal (N-terminus) labels. A fixed cysteine modification (C6H11NO, or 113.084) from the iST-NHS LYSE buffer was used, and methionine oxidation and N-terminal protein acetylation were set as variable modifications. Trypsin/P with up to two missed cleavages was used for the digestion mode. A reverse sequence database search was used for the target decoy search and to filter results on the peptide spectrum match (PSM) level to a 1% false discovery rate (FDR). Parameters were otherwise used at default values.

DIA-MS diaPASEF raw files from ScienCell cholangiocytes samples were converted to the HTRMS file format with HTRMS Convertor (v18.3), and then peptides were identified and peptide/protein MS intensities were quantified with Spectronaut (v18.6). Spectral libraries were computationally generated and searched against with the directDIA+ mode using the human reference proteome (UniProt proteome ID UP000005640, Swiss-Prot entries with isoforms, accessed 4 Jan 2021), using the default settings. In brief, a fixed carbamidomethyl cysteine modification was used, and methionine oxidation and N-terminal protein acetylation were set as variable modifications. Trypsin/P with up to two missed cleavages was used for the digestion mode. A “mutated” sequence database was used for the target decoy search to control results to the 1% FDR level at the PSM, peptide, and protein group levels, and cross-run normalization was enabled.

*Cell fixation and imaging with the U-2 OS GFP-LC3B autophagosome lysosome assay*

Fixation and washing were performed using batches of up to 5 plates per stacker on an EL406 plate washer/dispenser (BioTek, Winooski VT USA). The assay was scheduled so that each plate was fixed within 5 minutes after 3 hours of compound treatment. Cell fixation and staining of nuclei with HOECHST 33342 dye was carried out using a final concentration of 4% formaldehyde and 3.33 µg/mL HOECHST 33342 for 15 minutes at room temperature. Subsequently, the plates were stored at RT in the dark until image acquisition on the same day.

Images were acquired using an Opera Phenix (Revvity, Waltham MA USA) and image analysis was performed in Harmony 4.9/5.1 (Revvity). A 40x water-immersion objective was utilized in confocal mode to capture 16 fields of view per well in a stack of 5 planes, each with a distance of 1 µm. The assay generated three readouts: the number of nuclei, the number of GFP-LC3B (autophagosomes), and LysoTracker (lysosomes) spots. The number of healthy cells was determined based on the shape and intensity of HOECHST dye-stained nuclei. Small and bright nuclei, indicating condensed chromatin of dying cells, were excluded. A decrease in the number of healthy nuclei, indicating compound toxicity was reported alongside the readouts for autophagic flux: the number of LC3-spots per cell and lysosomes per cell.

*Stable Cas9 and dCas9 Cell Line Generation*

U-2 OS cells (ATCC HTB-96) were plated in a 6-well plate (3M cells/well) with 2 mL/well complete U-2 OS media: McCoy’s 5A media (Gibco 16600082) supplemented with 10% heat-inactivated FBS (Sigma F4135), 1% penicillin-streptomycin (Gibco 15140122), and a 1:1000 dilution of polybrene (Millipore TR-1003-G). Cells were then transduced with either 500 mL of lentivirus packaged with pLX-311-Cas9 - for CRISPR knockout (KO) screening – or dCas-VP64-Blast (pXPR-109) – for CRISPR activation (CRISPRa) screening- both obtained from the Broad Institute. The plate was spun at 2,000 RPM for 45 minutes before returning to the incubator. The following day, cells were lifted with 0.25% trypsin-EDTA, transferred to a 175 cm^2^ flask with complete fresh media and allowed to grow for three days. Following this, media was replaced with fresh media containing 10 mg/mL blasticidin (Sigma SBR00022) to select for transductants, and cells were grown for an additional five days. The surviving cells were then collected and cryopreserved for later screening.

*CRISPR gRNA Library Titrations*

CRISPR KO screening was performed with the human Brunello CRISPR-Cas9 knockout lentiviral library (80,000 gRNAs and four gRNAs per gene target) and CRISPRa screening was performed with the human Calabrese Set A activation lentiviral library (60,000 gRNAs and three gRNAs per gene target), obtained from the Broad Institute.

As both libraries contain a puromycin resistance marker, puromycin resistance was used as a surrogate for viral integration to determine a suitable library transduction volume. In brief, varying volumes of the respective lentiviral library (0, 5, 10, 25, 50, 75, 100 mL, etc) were added to a 6-well plate well containing 3M U-2 OS Cas9 (CRISPR KO) or U-2 OS dCas9 (CRISPRa) cells in 2 mL complete media. After cells were allowed to expand for three days, 0.016% of each cell population (corresponding to a particular library and transduction volume) was transferred to ten wells of 96-well plate well (100 mL final volume in media) and, the following day, cells were selected with (five wells) or without (five wells) 3 mg/mL puromycin. After three days of further incubation, the relative cell viability with and without puromycin selection, as seen using the Cell Titer Glo assay kit (Promega), was used to estimate the volume needed to obtain an approximate puromycin resistant population of 25%: 46 μL and 358 μL/well for Brunello CRISPR KO and Calabrese Set A CRISPRa libraries, respectively.

*CRISPR KO and CRISPRa transduction, selection with Compound X, and genomic DNA isolation*

Stable U-2 OS Cas9 (CRISPR KO) or U-2 OS dCas9 (CRISPRa) were expanded to >160M cells per line using 300 cm^2^ flasks before plating to multiple 6-well plates (3M cells/well in 2 mL complete U2-OS media). The volume of Brunello CRISPR KO and Calabrese Set A CRISPRa lentiviral library, determined from titration experiments, was added to each well of its corresponding cell line, and the plates were spun at 2,000 rpm for 45 min before overnight incubation. The following day, the wells for each cell line were divided by five, and pooled together to create five replicates, each containing >32M cells/replicate. Each pool was plated into 3x300 cm^2^ flasks (10M cells/flask) and incubated for three days, before repeating the puromycin titer test from above and replating to three fresh 300 cm^2^ flasks per pool (10M cells/flask). The following day, transductants in the flasks were selected for by addition of fresh media containing 3 mg/mL puromycin and an additional three days of incubation.

After the three-day puromycin selection step, the three flasks from each replicate were pooled together and sub-divided into 2x300 cm^2^ flask (10M cells/flask). The following day, media was replaced with fresh media containing 6 µM Compound X (CRISPR KO), 5 µM Compound X (CRISPRa), or DMSO (both screens), and incubated for three additional days. DMSO-treated flasks were passaged during the course of the experiment as needed, maintaining >10M cells per flask. After the treatment course, surviving cells were collected and duplicate treatment flasks within each replicate were pooled together. DNA was isolated using Qiagen Puregene Kits following the manufacturer protocol.

*NGS Library Preparation and DNA Sequencing*

To avoid undersampling of the lentiviral vector libraries, 12x100 mL PCR reactions were performed per each sample. Each reaction contained: 10 mL PCR buffer (Clontech Takara 639242), 8 mL dNTPs (Clontech Takara 4030), 5 mL DMSO (Sigma D2650), 0.5 mL 5 mM P7 primers (Integrated DNA Technologies, IDT), 0.5 mL 100 mM P5 primer (IDT), 1.5 mL Titanium *Taq* DNA Polymerase, up to 10 μg of purified DNA, and nuclease free water. P5 is a pool of eight primers mixed at equal molarity and P7 primers contain sample unique barcodes used for demultiplexing (**Table S1**). PCR reactions were amplified with the following thermocycler program: 95 °C for 5 minutes; 28 cycles of 95 °C for 30 seconds, 53 °C for 30 seconds, and 72 °C for 20 seconds; 72 °C for 10 minutes; hold at 4 °C.

PCR wells containing identical reactions were pooled together into a single 1.5 mL Eppendorf tube and purified using the AMPure XP-PCR purification kit (Beckman Coulter A63880), with a final elution in 30-50 mL TE buffer and stored at -20 °C until shipment to the Novogene Corporation (Sacramento, CA) for sequencing. After demultiplexing of sequencing data, reads with guide RNA sequences were extracted and normalized, and analyzed for guide and gene level effects as previously described^44^.

**Supplementary Note**
An enrichment analysis of proteins with significant increases following Compound X treatment as the Reactome entry “Post-translational protein phosphorylation” (R-HSA-8957275) as one of the top two enriched pathways. This pathway contains secreted proteins that are substrates of the secretory protein kinase FAM20C; the protein hits contributing to this enrichment were APOB, C4A, APLP2, MXRA8, and TMEM132A.

Interestingly, a careful inspection of the tandem mass tag (TMT) mass spectrometry data for ABM and Creative Bioarray cholangiocytes revealed that the protein with the greatest increase following Compound X treatment (log_2_FC = 3.08 for 3 μM vs. vehicle, adjusted *p* value < 1e-06) was a non-human protein that was included in the MS data search via the MaxQuant software package’s built-in list of common experimental contaminants. This protein (ENSBTAP00000034412) was bovine apolipoprotein B (APOB) and was quantified via a greater number of unique, high confidence peptide identifications than were for identified for the separate human APOB entry (25 and 6 peptides, respectively).

Data-independent acquisition mass spectrometry (DIA-MS) of ScienCell cholangiocytes also identified APOB as a protein with a significant increase following Compound X treatment (log_2_FC = 2.31 for 3 µM vs. vehicle, adjusted *p* value < 1e-07), a protein-level quantification derived from high confidence identifications of 20 tryptic peptides matching human APOB (UniProt ID P04114) uniquely within the human proteome.

As the Spectronaut software package does not search against common non-human protein contaminants, we did a second spectral library search of the same files against the bovine proteome (UniProt proteome ID UP000009136, Swiss-Prot entries with isoforms, accessed 29 May 2024). This search also identified bovine APOB with significant increases following Compound X treatment (log_2_FC = 2.28, adjusted *p* value < 1e-06 for 3 µM vs. vehicle), but now derived from high confidence identifications of 237 tryptic peptides matching bovine APOB (UniProt ID E1BNR0) uniquely within the bovine proteome.

Moreover, an alignment of the human and bovine APOB sequences revealed that of the 20 peptides originally attributed to human APOB, 12 perfectly matched both the bovine and human sequences, despite moderate cross-species identity in the primary amino acid sequence (73.8%) and their tryptic peptides (44 out of 366 for peptides of length four or more).

The greater number of APOB peptides which match the bovine sequence, exceeding the total number of tryptic peptide sequences that are shared between human and bovine APOB, argues that the majority of peptides identified in these experiments are of bovine origin, namely fetal bovine serum used in cell culture. As the cells were washed three times with phosphate buffer serum during harvesting, and the bovine APOB protein quantification increases with Compound X treatment, this suggests that bovine serum APOB is taken up by cholangiocytes, and that the degrader either increases this uptake or inhibits subsequent lysosomal degradation.

Similar observations were made for another increasing protein “hit”, Complement C4-A (13 tryptic peptides matching the human sequence P0C0L4; 58 matching the bovine sequence A0AAA9S3H4). However, identifications for the amyloid beta precursor like protein 2 (ABLP2) better matched the human sequence (24 tryptic peptides matching the human sequence P0C0L4; 11 matching the bovine sequence A0A3Q1M9W8), demonstrating that not all affected secreted proteins are necessarily derived from bovine serum.

**Supplementary Figures**

**
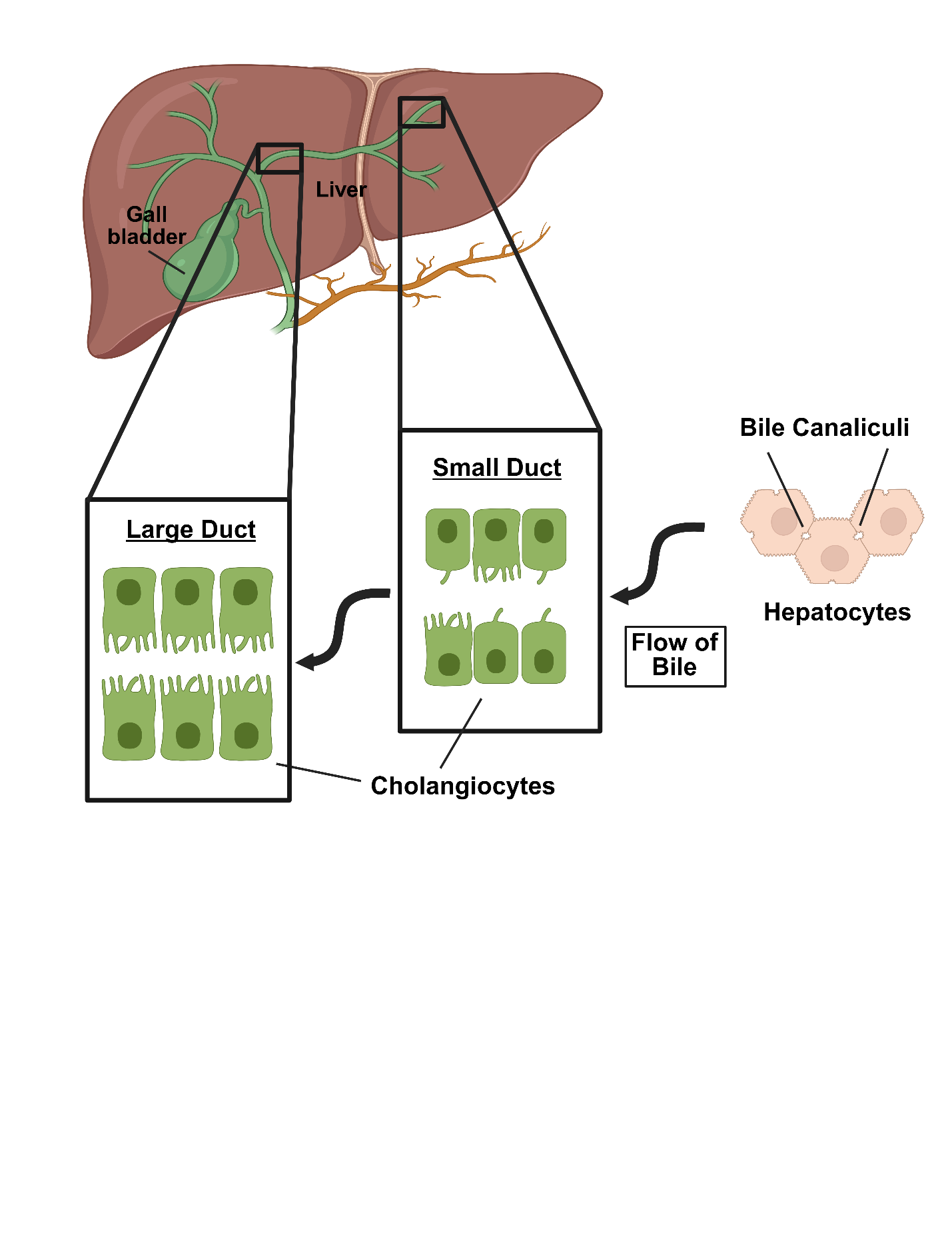
**

**Figure S1.** Anatomic illustration of the primary components of the hepatobiliary system. A macroscopic reference of the liver and extrahepatic (outside of the liver) and intrahepatic (inside the liver) biliary tree is provided. Large ducts are those extra- and intra-hepatic ducts of relatively large diameter proximate to the gall bladder and common bile duct. Small ducts branch from the large ducts and intercalate within the hepatic lobules. Following the flow of bile: hepatocyte-produced bile components filter into the inter-hepatic bile canaliculi, pass through the hepatic lobule to the small ducts, which then converge on the large ducts as bile flow progresses toward the gall bladder. This figure was created with BioRender.com.


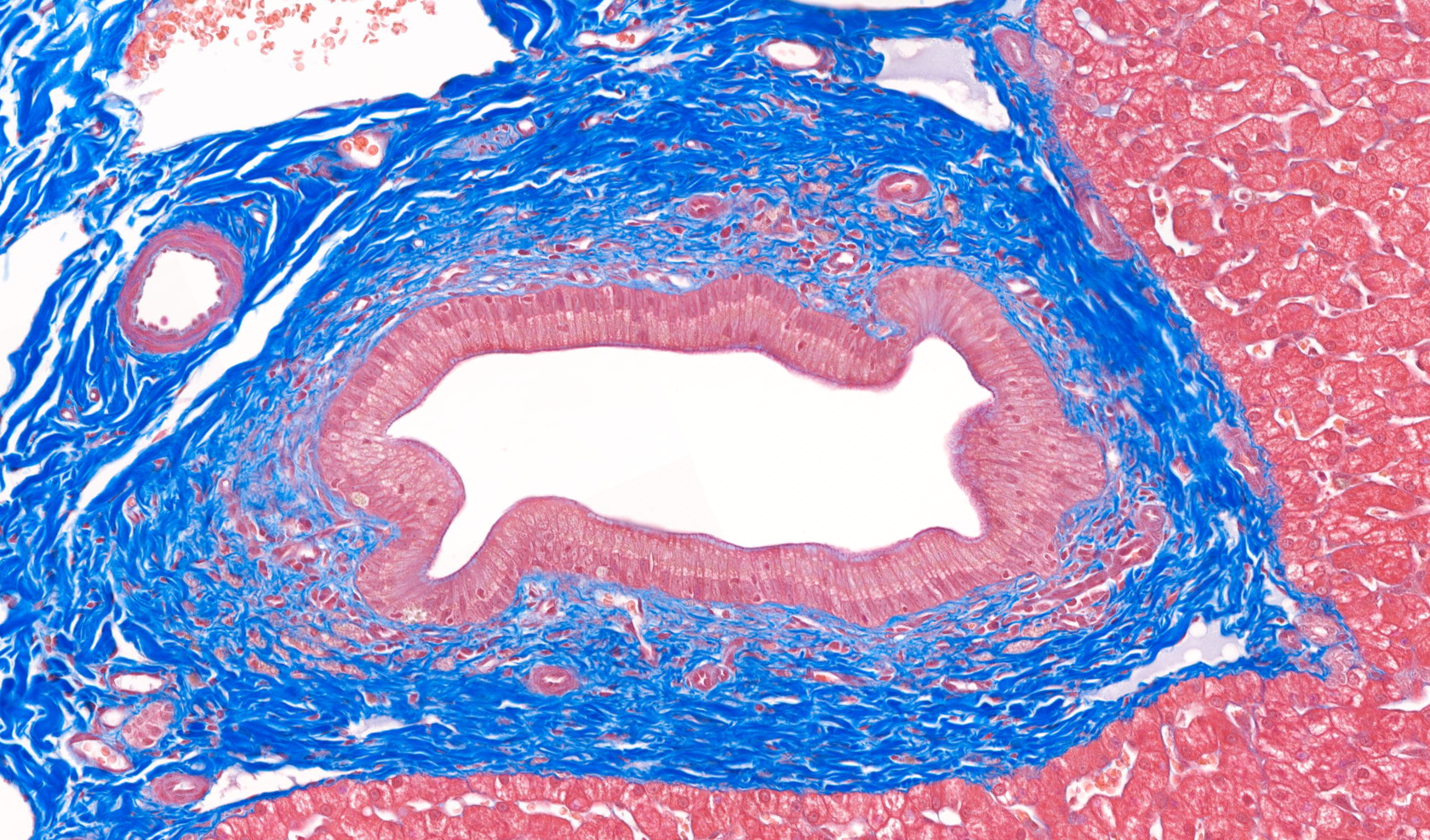

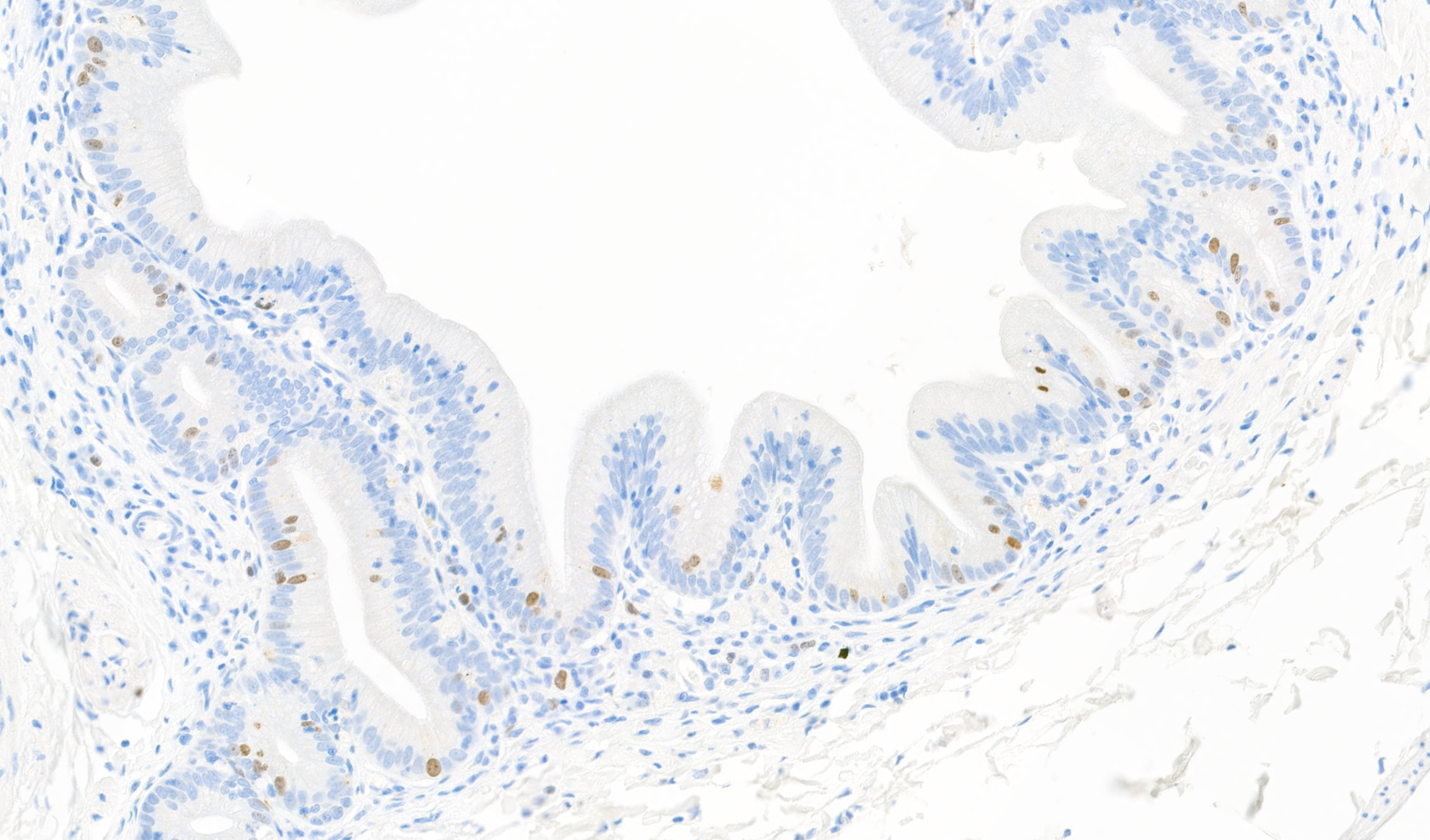

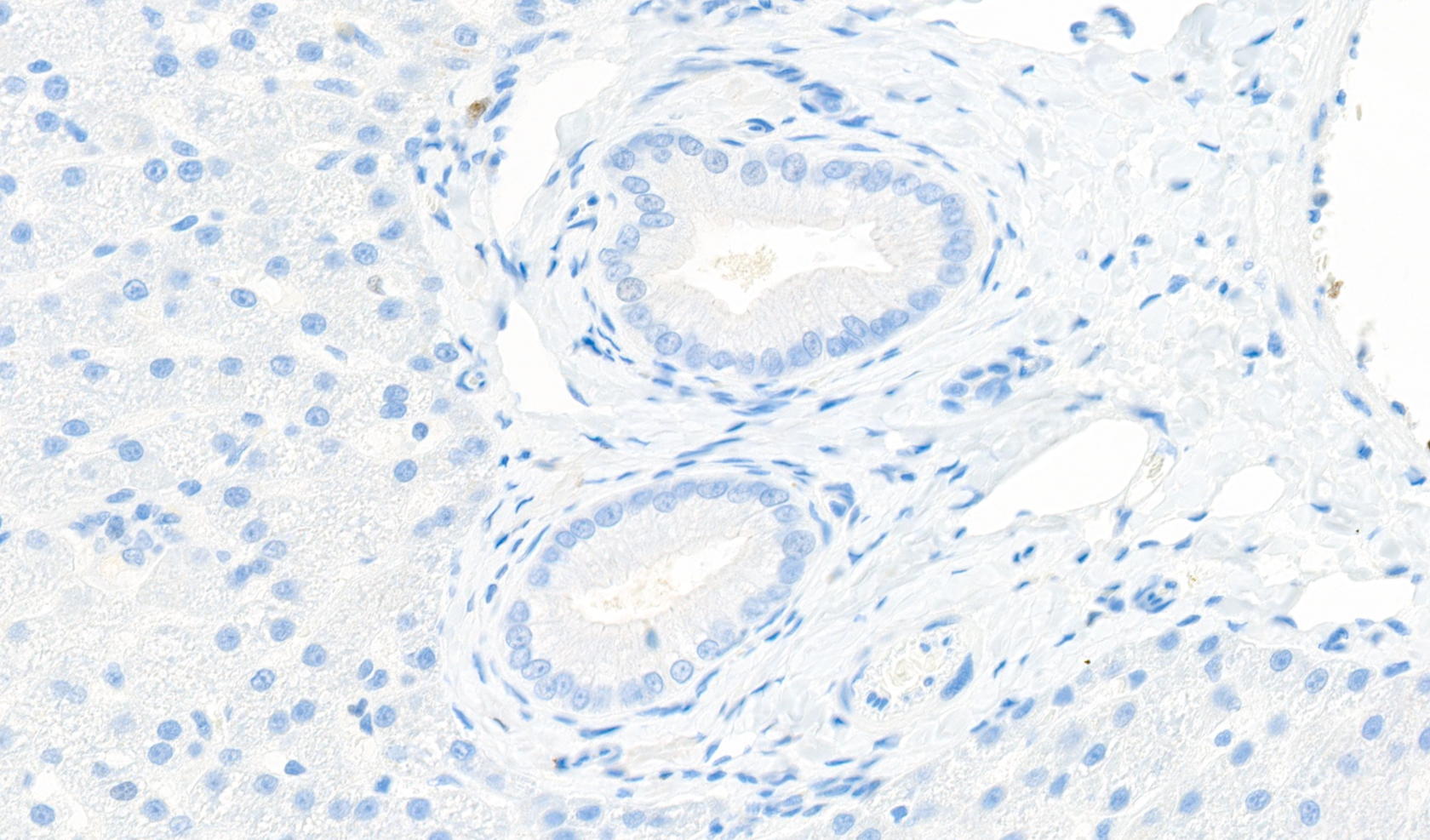


**(A)**


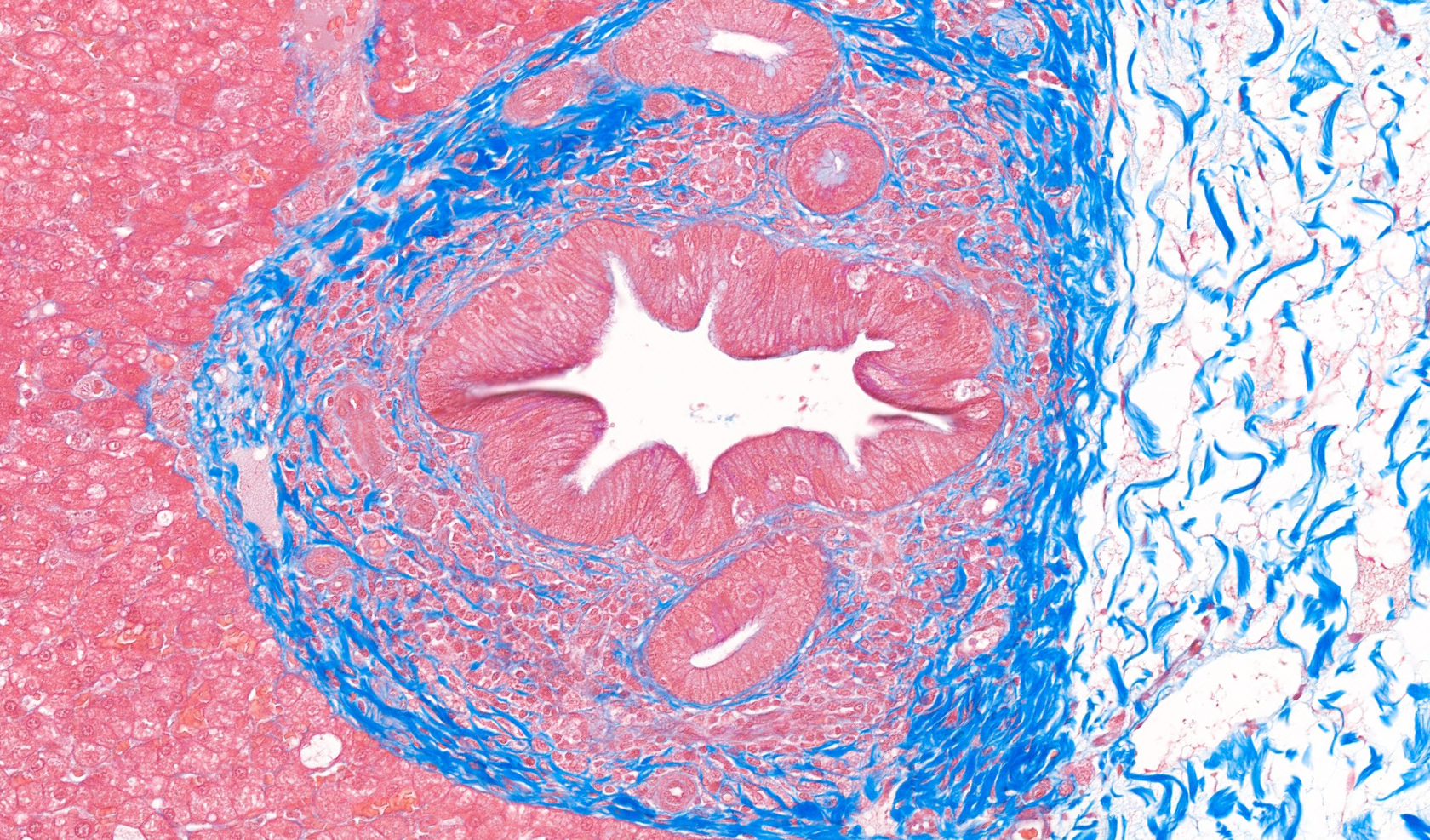


**(B)**


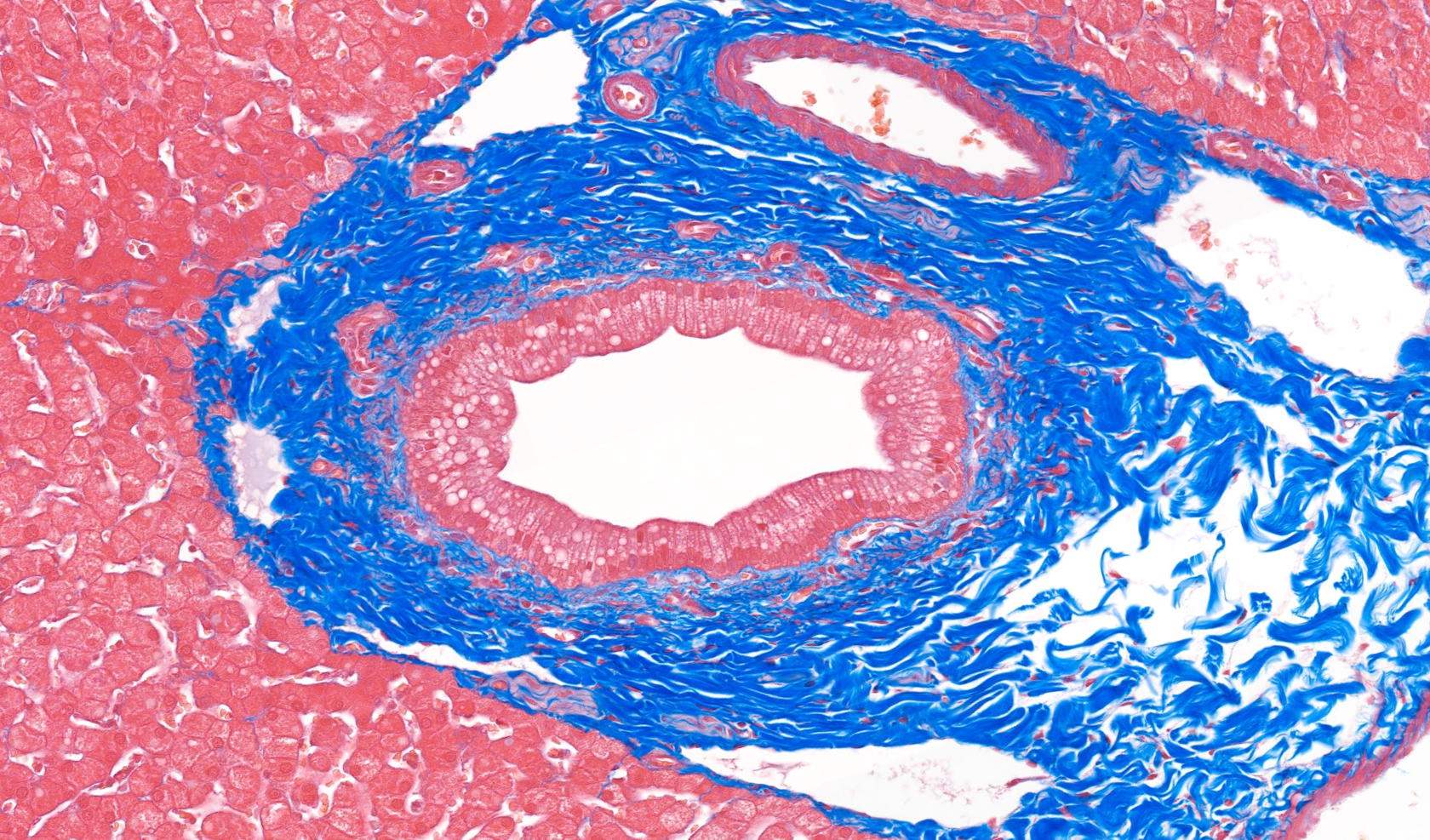


*

*

**(C)**

*

*

*

**Figure S2.** Administration of Compound X results in biliary hyperplasia and peri-ductular fibrosis in dogs. (A) Increased Ki67-positive nuclear staining (arrows) in cholangiocytes in a dog administered 30 mg/kg/day Compound X for 4 weeks, Ki67 IHC, 20X. Inset: Large bile duct from a control dog, Ki67 IHC, 20X. (B-C) Periductal fibrosis (asterisks) in a dog administered 30 mg/kg/day Compound X for 4 weeks (B, inset: control dog) and after 4 weeks of recovery (C) consistent with an irreversible change. Masson’s trichrome stain, 20X.

**
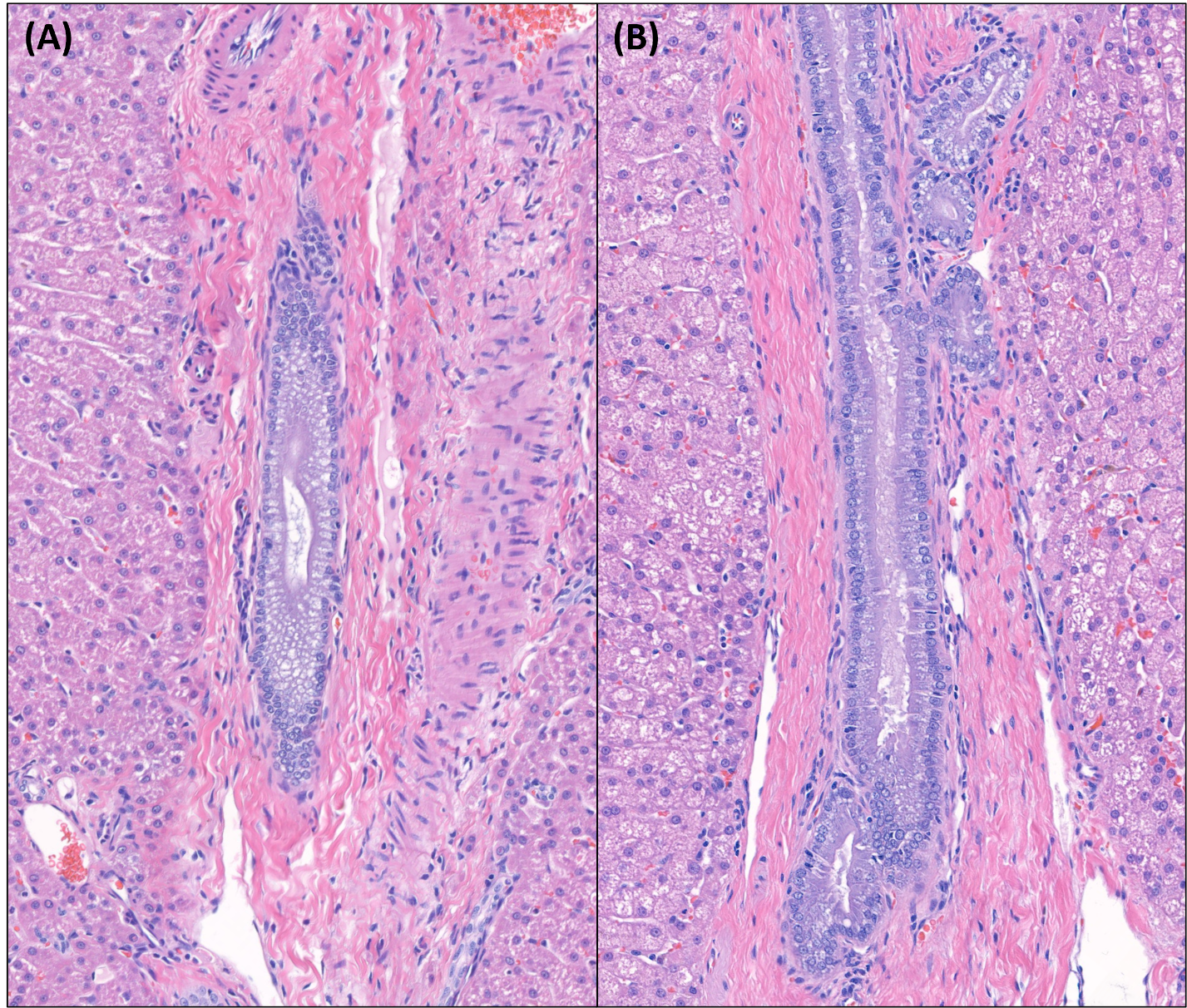
**

**Figure S3.** No significant histologic findings in the large bile duct of dogs administered Compound Y (A) or Compound Z (B) for 5 days, HE, 20X.

**
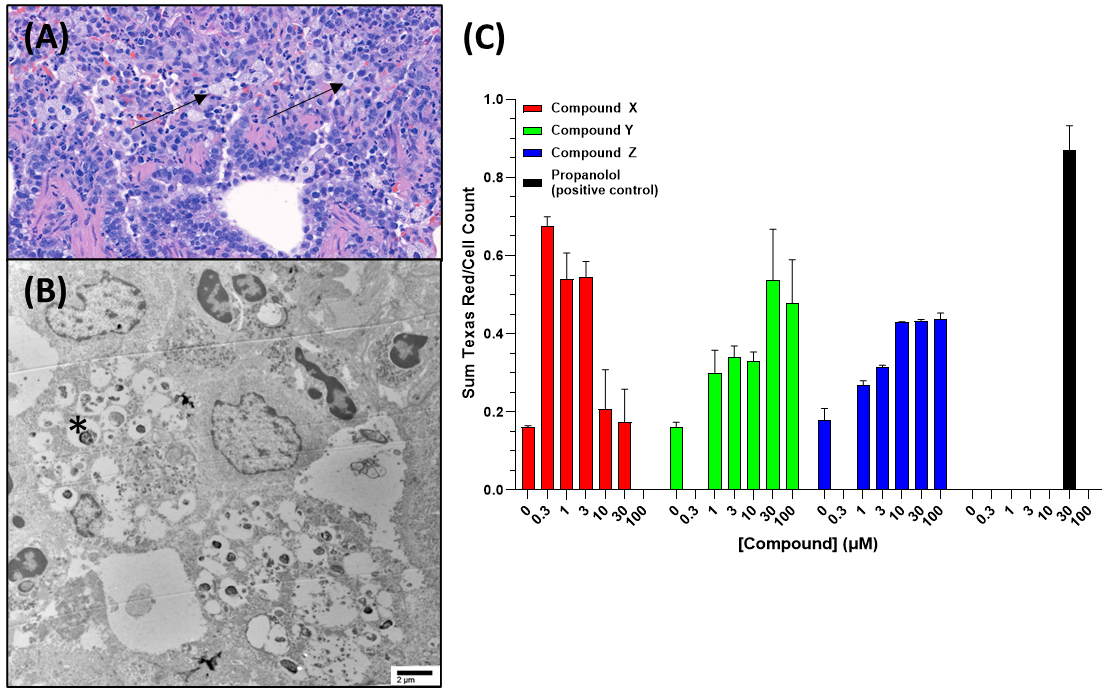

Figure S4.** Basic and lipophilic degraders may cause phospholipidosis. (A) 5- day administration of Compound Z resulted moderate mixed inflammation in the lung with foamy macrophages (arrows), HE, 20X. (B) Ultrastructural imaging revealed intracellular vacuoles containing membrane whorls (asterisk) consistent with phospholipidosis. (C) Phospholipidosis assay in HepG2 cells shows dose-dependent induction of Texas Red intensity for all three degrader compounds. Note loss of viability in HepG2 with Compound X at and above 10 μM. Propanol 1 is used as a positive control.

**
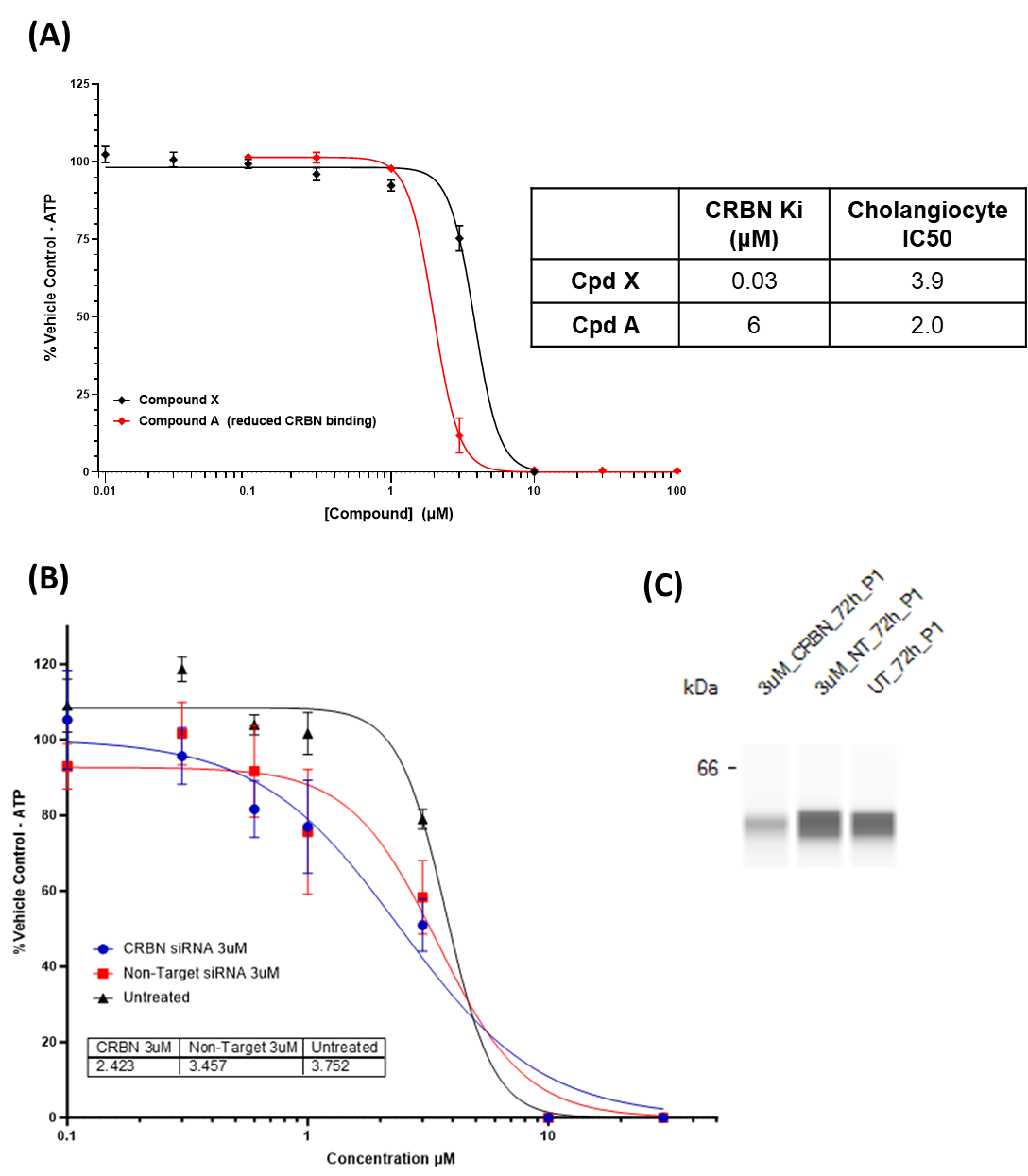
Figure S5.** CRBN is dispensable for Compound X-mediated cytotoxicity in human cholangiocytes. (A) Cholangiocyte cytotoxicity is equivalent for Compound X and inactive compound Compound A despite 200-fold less binding to CRBN; (B) Cholangiocyte cytotoxicity is equivalent for cells transfected with siRNA against CRBN; (C) Confirmation of CRBN knockdown by Western blot.

**Figure S6.** Compound X shows cholestatic potential at 10 μM and cytotoxicity to primary human hepatocytes above 30 μM. Neither Compound Y nor Compound Z show any impact on hepatocyte viability.

**
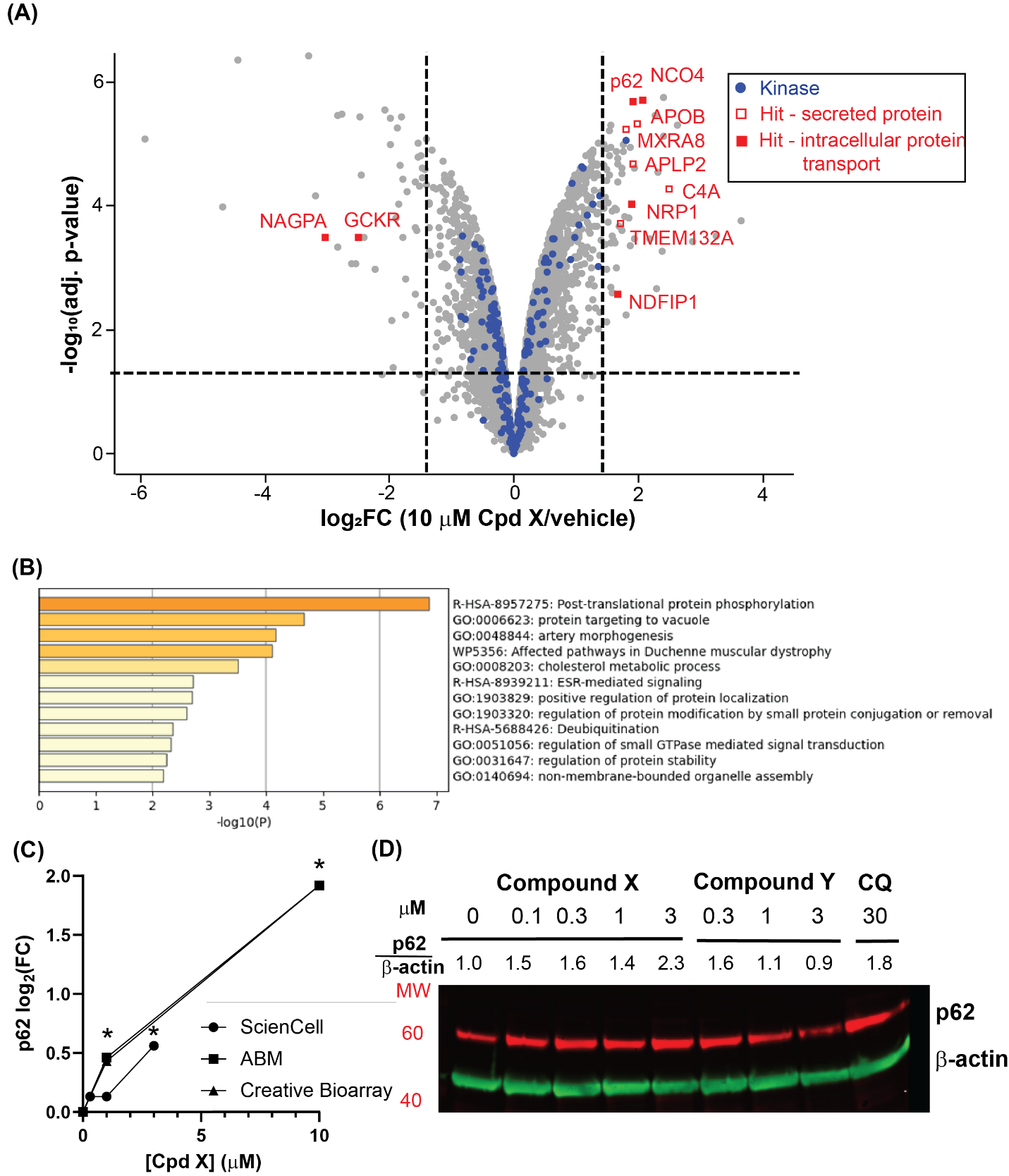
Figure S7.** Proteomics of cholangiocytes treated with Compound X (0.3 – 10 µM) for 18 hr reveals increases in secreted proteins and proteins involves in intracellular transport. (A) A representative volcano plot of protein abundance changes quantified in cholangiocytes (Applied Biological Materials) treated with 10 µM Compound X. (B) Enrichment analysis using Metascape of significantly changing proteins. Proteins from the top two categories are labeled in red and blue. (C) Concentration-dependent protein level changes of p62 (SQSTM1) in cholangiocytes from three vendors. An asterisk represents a protein abundance change that is significant (adjusted *p* value < 0.05). (D) Compound X but not Compound Y induces a concentration-dependent increase in p62 abundance in primary cholangiocytes after 18h treatment, as determined by immunoblot.

**
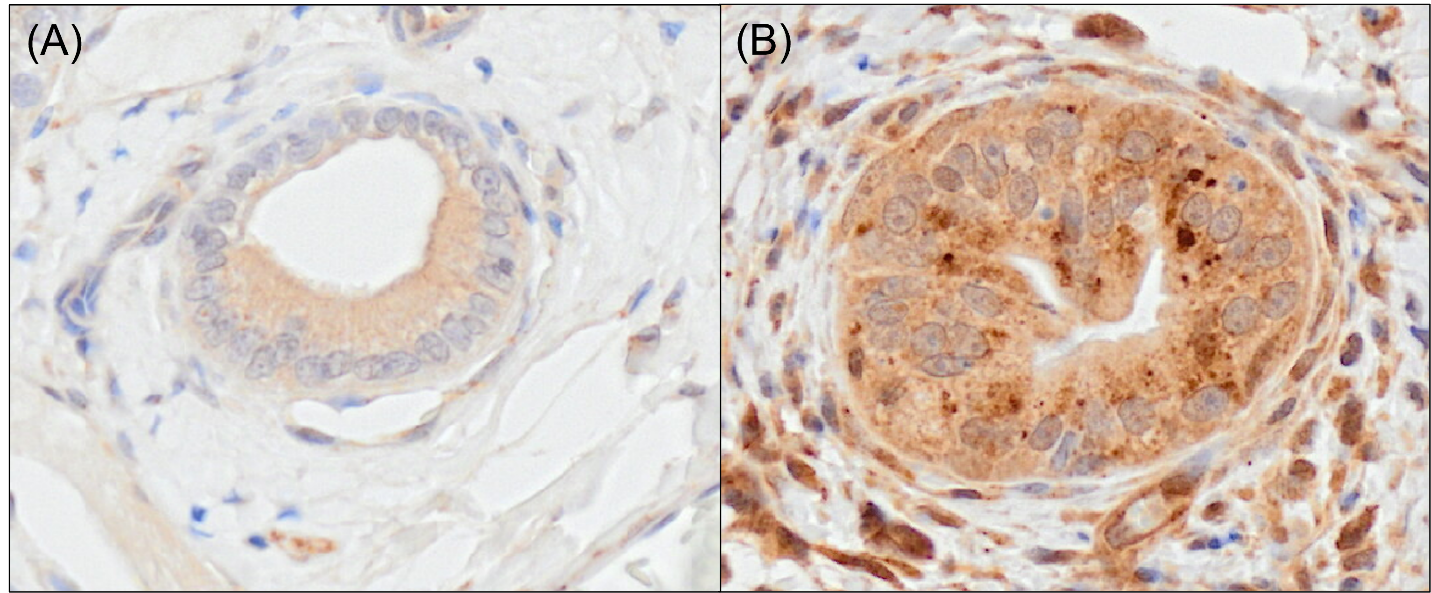
**

**Figure S8.** Representative photomicrograph of bile ducts in dogs. (A) Control dog. (B) Compound X at 30 mg/kg/day for 5 days. Note dense to punctate brown staining consistent with nucleation of LC3B. LC3B IHC, 20X.

**
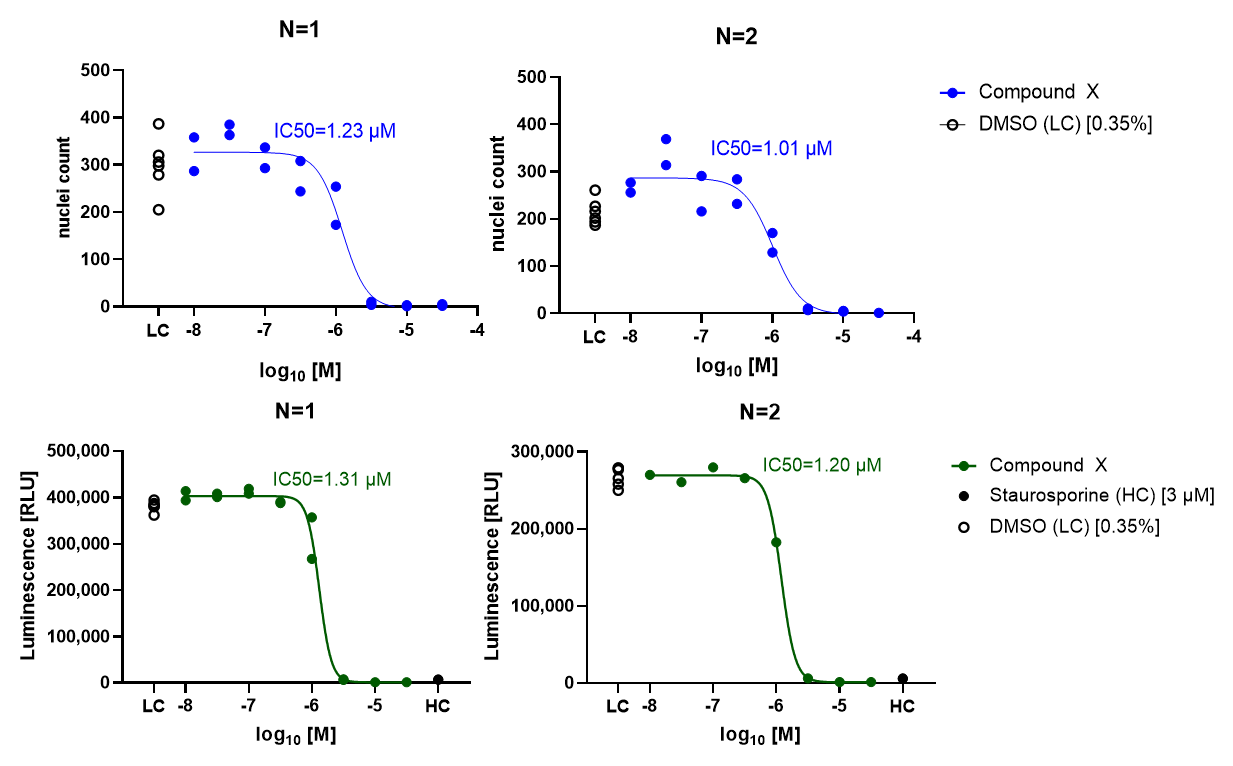
**

**Figure S9.** Cell viability of U-2 OS GFP-LC3B cells treated for 48 h with Compound X as measured by (A) nuclei count by Hoesct staining and image analysis or (B) ATP quantification by Cell Titer Glo viability assay.

**
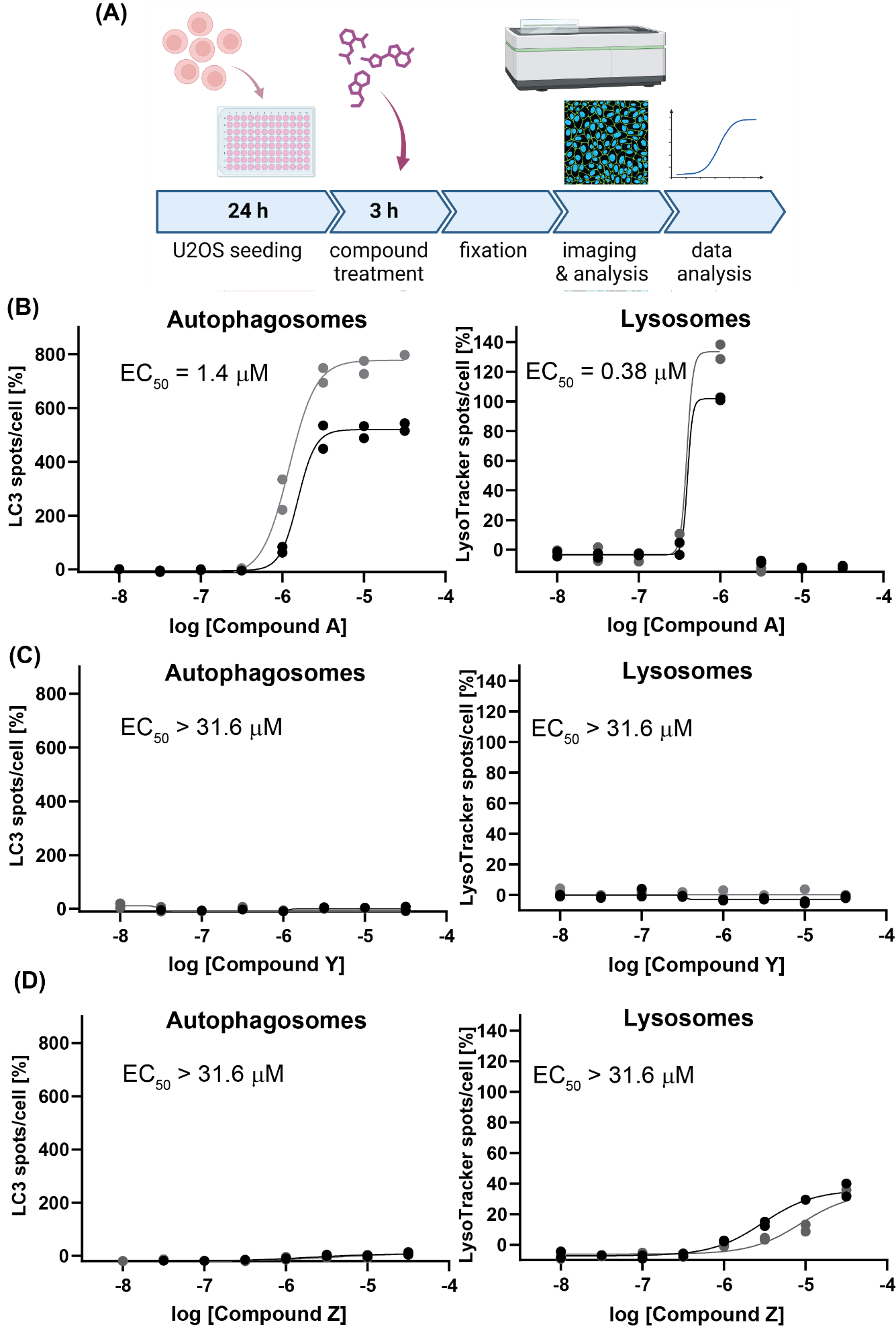

Figure S10.** (A) Schematic of the high content imaging of U-2 OS GFP-LC3B cells with data for (B) Compound A, (C) Compound Y, and (D) Compound Z. Compound A treatment, which does not bind CRBN, is followed by similar induction of autophagosome number and biphasic effect for lysosomes as Compound X; Compound Y and Compound Z induce minimal changes. EC50 values displayed represent the geometric mean of values from the two replicates shown; reduced LysoTracker values for Compound A at >1 μM were excluded from fits.

**
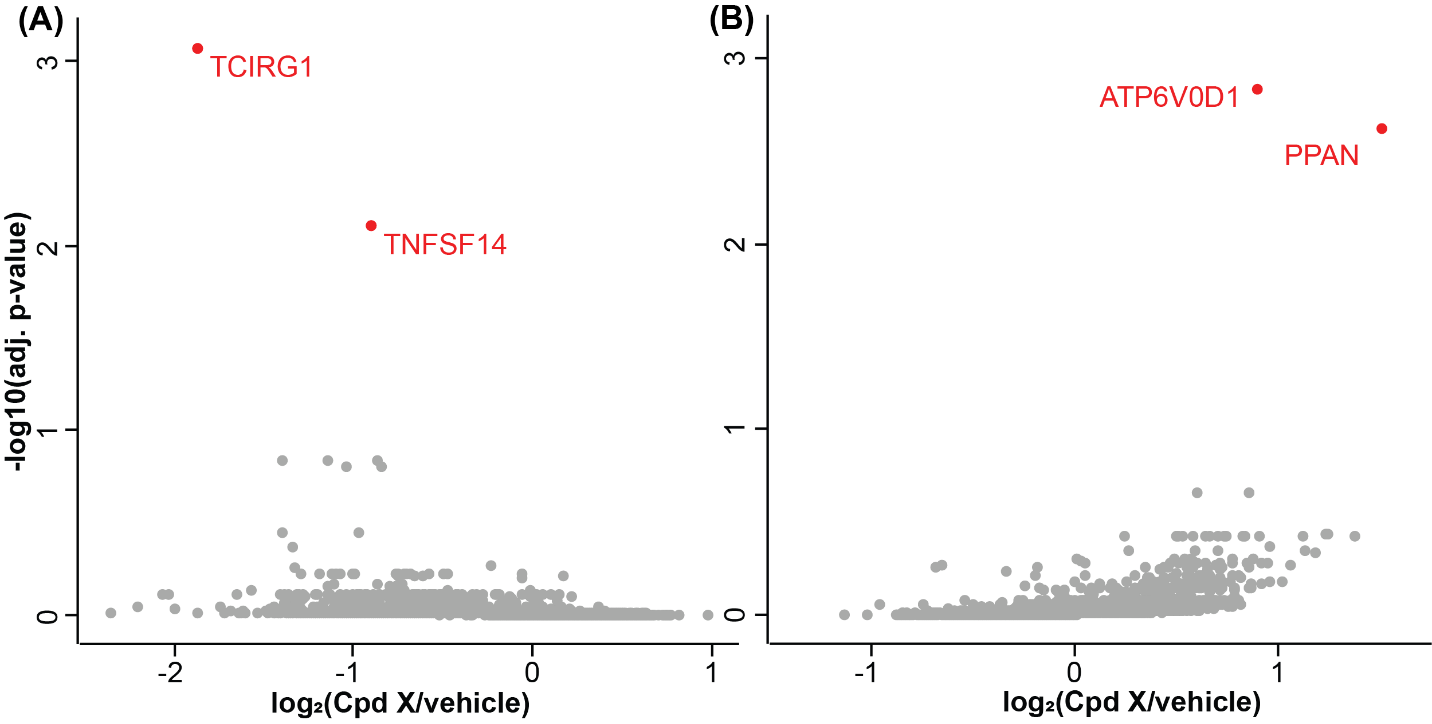
Figure S11.** Volcano plots displaying median log_2_FC effects and adjusted *p* value significance for genes that are (A) enriched upon Compound X treatment in a CRISPR KO screen or (B) depleted upon Compound X treatment in a CRISPRa screen.

**Supplementary Tables**

**Table S1**

| P5 ARGON #1 | AATGATACGGCGACCACCGAGATCTACACTCTTTCCCTACACGACGCTCTTCCGATCTTTGTGGAAAGGACGAAAC*A*C*C*G |
| --- | --- |
| P5 ARGON #2 | AATGATACGGCGACCACCGAGATCTACACTCTTTCCCTACACGACGCTCTTCCGATCTCTTGTGGAAAGGACGAAAC*A*C*C*G |
| P5 ARGON #3 | AATGATACGGCGACCACCGAGATCTACACTCTTTCCCTACACGACGCTCTTCCGATCTGCTTGTGGAAAGGACGAAAC*A*C*C*G |
| P5 ARGON #4 | AATGATACGGCGACCACCGAGATCTACACTCTTTCCCTACACGACGCTCTTCCGATCTAGCTTGTGGAAAGGACGAAAC*A*C*C*G |
| P5 ARGON #5 | AATGATACGGCGACCACCGAGATCTACACTCTTTCCCTACACGACGCTCTTCCGATCTCAACTTGTGGAAAGGACGAAAC*A*C*C*G |
| P5 ARGON #6 | AATGATACGGCGACCACCGAGATCTACACTCTTTCCCTACACGACGCTCTTCCGATCTTGCACCTTGTGGAAAGGACGAAAC*A*C*C*G |
| P5 ARGON #7 | AATGATACGGCGACCACCGAGATCTACACTCTTTCCCTACACGACGCTCTTCCGATCTACGCAACTTGTGGAAAGGACGAAAC*A*C*C*G |
| P5 ARGON #8 | AATGATACGGCGACCACCGAGATCTACACTCTTTCCCTACACGACGCTCTTCCGATCTGAAGACCCTTGTGGAAAGGACGAAAC*A*C*C*G |

| P7: Calabrese 5 μM Drug Treated Rep 1 | CAAGCAGAAGACGGCATACGAGATGCACGACCGTGACTGGAGTTCAGACGTGTGCTCTTCCGATCTTCTACTATTCTTTCCCCTGCA*C*T*G*T |
| --- | --- |
| P7: Calabrese 5 μM Drug Treated Rep 2 | CAAGCAGAAGACGGCATACGAGATATGTCTCCGTGACTGGAGTTCAGACGTGTGCTCTTCCGATCTTCTACTATTCTTTCCCCTGCA*C*T*G*T |
| P7: Calabrese 5 μM Drug Treated Rep 3 | CAAGCAGAAGACGGCATACGAGATATACTCGGGTGACTGGAGTTCAGACGTGTGCTCTTCCGATCTTCTACTATTCTTTCCCCTGCA*C*T*G*T |
| P7: Calabrese 5 μM Drug Treated Rep 4 | CAAGCAGAAGACGGCATACGAGATCGCAGACCGTGACTGGAGTTCAGACGTGTGCTCTTCCGATCTTCTACTATTCTTTCCCCTGCA*C*T*G*T |
| P7: Calabrese 5 μM Drug Treated Rep 5 | CAAGCAGAAGACGGCATACGAGATTACACTCCGTGACTGGAGTTCAGACGTGTGCTCTTCCGATCTTCTACTATTCTTTCCCCTGCA*C*T*G*T |
| P7: Calabrese DMSO Treated Rep 1 | CAAGCAGAAGACGGCATACGAGATCGTGAGCCGTGACTGGAGTTCAGACGTGTGCTCTTCCGATCTTCTACTATTCTTTCCCCTGCA*C*T*G*T |
| P7: Calabrese DMSO Treated Rep 2 | CAAGCAGAAGACGGCATACGAGATGCGTAGCCGTGACTGGAGTTCAGACGTGTGCTCTTCCGATCTTCTACTATTCTTTCCCCTGCA*C*T*G*T |
| P7: Calabrese DMSO Treated Rep 3 | CAAGCAGAAGACGGCATACGAGATGCCAAGTTGTGACTGGAGTTCAGACGTGTGCTCTTCCGATCTTCTACTATTCTTTCCCCTGCA*C*T*G*T |
| P7: Calabrese DMSO Treated Rep 4 | CAAGCAGAAGACGGCATACGAGATATTGGATTGTGACTGGAGTTCAGACGTGTGCTCTTCCGATCTTCTACTATTCTTTCCCCTGCA*C*T*G*T |
| P7: Calabrese DMSO Treated Rep 5 | CAAGCAGAAGACGGCATACGAGATCGACTCTTGTGACTGGAGTTCAGACGTGTGCTCTTCCGATCTTCTACTATTCTTTCCCCTGCA*C*T*G*T |
| P7: Brunello 6 μM Drug Treated Rep 1 | CAAGCAGAAGACGGCATACGAGATTAGTTCGGGTGACTGGAGTTCAGACGTGTGCTCTTCCGATCTTCTACTATTCTTTCCCCTGCA*C*T*G*T |
| P7: Brunello 6 μM Drug Treated Rep 2 | CAAGCAGAAGACGGCATACGAGATCGCAAGTTGTGACTGGAGTTCAGACGTGTGCTCTTCCGATCTTCTACTATTCTTTCCCCTGCA*C*T*G*T |
| P7: Brunello 6 μM Drug Treated Rep 3 | CAAGCAGAAGACGGCATACGAGATTATGTCAAGTGACTGGAGTTCAGACGTGTGCTCTTCCGATCTTCTACTATTCTTTCCCCTGCA*C*T*G*T |
| P7: Brunello 6 μM Drug Treated Rep 4 | CAAGCAGAAGACGGCATACGAGATGCGTAGTTGTGACTGGAGTTCAGACGTGTGCTCTTCCGATCTTCTACTATTCTTTCCCCTGCA*C*T*G*T |
| P7: Brunello 6 μM Drug Treated Rep 5 | CAAGCAGAAGACGGCATACGAGATTAGTAGAAGTGACTGGAGTTCAGACGTGTGCTCTTCCGATCTTCTACTATTCTTTCCCCTGCA*C*T*G*T |
| P7: Brunello DMSO Treated Rep 1 | CAAGCAGAAGACGGCATACGAGATTAGTCTAAGTGACTGGAGTTCAGACGTGTGCTCTTCCGATCTTCTACTATTCTTTCCCCTGCA*C*T*G*T |
| P7: Brunello DMSO Treated Rep 2 | CAAGCAGAAGACGGCATACGAGATCGGTAGCCGTGACTGGAGTTCAGACGTGTGCTCTTCCGATCTTCTACTATTCTTTCCCCTGCA*C*T*G*T |
| P7: Brunello DMSO Treated Rep 3 | CAAGCAGAAGACGGCATACGAGATCGACAGCCGTGACTGGAGTTCAGACGTGTGCTCTTCCGATCTTCTACTATTCTTTCCCCTGCA*C*T*G*T |
| P7: Brunello DMSO Treated Rep 4 | CAAGCAGAAGACGGCATACGAGATCGGTGAGGGTGACTGGAGTTCAGACGTGTGCTCTTCCGATCTTCTACTATTCTTTCCCCTGCA*C*T*G*T |
| P7: Brunello DMSO Treated Rep 5 | CAAGCAGAAGACGGCATACGAGATCGACAGTTGTGACTGGAGTTCAGACGTGTGCTCTTCCGATCTTCTACTATTCTTTCCCCTGCA*C*T*G*T |

**Supplementary References**

(43) Meier, F.; Brunner, A.-D.; Frank, M.; Ha, A.; Bludau, I.; Voytik, E., *et al*. DiaPASEF: Parallel Accumulation–Serial Fragmentation Combined with Data-Independent Acquisition. *Nat. Methods* **2020**, *17* (12), 1229–1236. <https://doi.org/10.1038/s41592-020-00998-0>.

(44) Petiwala, S.; Modi, A.; Anton, T.; Murphy, E.; Kadri, S.; Hu, H., *et al*. *CRISPR J.* **2023**, *6* (1), 75–82. <https://doi.org/10.1089/crispr.2022.0093>.
